# Modeling of lung phenotype of Hermansky–Pudlak syndrome type I using patient-specific iPSCs

**DOI:** 10.1186/s12931-021-01877-8

**Published:** 2021-11-04

**Authors:** Takahiro Suezawa, Shuhei Kanagaki, Yohei Korogi, Kazuhisa Nakao, Toyohiro Hirai, Koji Murakami, Masatoshi Hagiwara, Shimpei Gotoh

**Affiliations:** 1 Department of Drug Discovery for Lung Diseases, Graduate School of Medicine, Kyoto University, Yoshida Konoe-cho, Sakyo-ku, Kyoto, 606-8501 Japan; 2 Watarase Research Center, Kyorin Pharmaceutical Co. Ltd., Shimotsuga-gun, Tochigi, Japan; 3 Department of Respiratory Medicine, Graduate School of Medicine, Kyoto University, Kyoto, Japan; 4 Department of Anatomy and Developmental Biology, Graduate School of Medicine, Kyoto University, Kyoto, Japan

**Keywords:** HPS1, Patient-specific iPSCs, Alveolar epithelial type 2 cells, Giant lamellar body

## Abstract

**Background:**

Somatic cells differentiated from patient-specific human induced pluripotent stem cells (iPSCs) could be a useful tool in human cell-based disease research. Hermansky–Pudlak syndrome (HPS) is an autosomal recessive genetic disorder characterized by oculocutaneous albinism and a platelet dysfunction. HPS patients often suffer from lethal HPS associated interstitial pneumonia (HPSIP). Lung transplantation has been the only treatment for HPSIP. Lysosome-related organelles are impaired in HPS, thereby disrupting alveolar type 2 (AT2) cells with lamellar bodies. HPSIP lungs are characterized by enlarged lamellar bodies. Despite species differences between human and mouse in HPSIP, most studies have been conducted in mice since culturing human AT2 cells is difficult.

**Methods:**

We generated patient-specific iPSCs from patient-derived fibroblasts with the most common bi-allelic variant, c.1472_1487dup16, in HPS1 for modeling severe phenotypes of HPSIP. We then corrected the variant of patient-specific iPSCs using CRISPR-based microhomology-mediated end joining to obtain isogenic controls. The iPSCs were then differentiated into lung epithelial cells using two different lung organoid models, lung bud organoids (LBOs) and alveolar organoids (AOs), and explored the phenotypes contributing to the pathogenesis of HPSIP using transcriptomic and proteomic analyses.

**Results:**

The LBOs derived from patient-specific iPSCs successfully recapitulated the abnormalities in morphology and size. Proteomic analysis of AOs involving iPSC-derived AT2 cells and primary lung fibroblasts revealed mitochondrial dysfunction in *HPS1* patient-specific alveolar epithelial cells. Further, giant lamellar bodies were recapitulated in patient-specific AT2 cells.

**Conclusions:**

The HPS1 patient-specific iPSCs and their gene-corrected counterparts generated in this study could be a new research tool for understanding the pathogenesis of HPSIP caused by *HPS1* deficiency in humans.

**Supplementary Information:**

The online version contains supplementary material available at 10.1186/s12931-021-01877-8.

## Background

Advancements in stem cell biology have made it possible to induce differentiation of pluripotent stem cells to obtain various human somatic cells, including those that are difficult to obtain from human tissues [1]. Human induced pluripotent stem cells (iPSCs) are not extracted from human blastocysts, and thus, are considered more ethical than human embryonic stem cells (ESCs). Easily accessible somatic cells, such as skin fibroblasts or peripheral blood cells, can be reprogramed to establish iPSCs with patient-specific genetic backgrounds [2, 3]. iPSCs can be stocked and are expected to be useful resources for studies on diseases, wherein somatic cells differentiated from iPSCs can be used to recapitulate disease phenotypes. Although the scope for applications of human iPSCs is still limited, evidence suggests that iPSC-based disease models are suitable for understanding the disease mechanisms and drug discovery in humans [4].

Hermansky–Pudlak syndrome (HPS) is an autosomal recessive disorder characterized by oculocutaneous albinism and a platelet dysfunction [5]. Mutations in HPS causative genes induce abnormal biogenesis of lysosome-related organelles (LROs) by impairing membrane trafficking [6]. Out of the 11 HPS causative genes, mutations in *HPS1*, *AP3B1*, and *HPS4* can induce lethal HPS-associated interstitial pneumonia (HPSIP), for which lung transplantation is the only treatment available [5, 7, 8]. Mutations in *HPS1* are the most common in HPS, worldwide. HPS has high prevalence in Puerto Rico, with the c.1472_1487dup16 variant of *HPS1* accounting for 45% of the cases [9]. Patients with loss-of-function *HPS1* mutations, including c.1472_1487dup16, show severe symptoms, with full penetrance of HPSIP in the middle age [7]. HPSIP is irreversible progressive interstitial fibrosis similar to idiopathic pulmonary fibrosis (IPF); however its HPSIP onset occurs earlier than IPF [10]. One of the salient histopathologic features of HPSIP is the abnormal enlargement of lamellar bodies, which are LROs that store pulmonary surfactant in alveolar type 2 (AT2) cells [11]. Abnormalities in a variety of cell types were observed in HPSIP [12–14]; however, HPS mouse models have shown that vulnerability of AT2 cells could be primarily responsible for HPSIP development [15, 16]. Most HPSIP studies have used mice because obtaining and culturing primary AT2 cells derived from HPS patients’ lungs is difficult. However, there are species-specific differences between HPSIP in humans and mice [7]. Therefore, human pluripotent stem cells could be a cell source. Recently, HPSIP models using ESC-derived lung bud organoids (LBOs) were reported; however, they did not represent the alveolar epithelial cell lineage with lamellar bodies, the key organelle in HPSIP [17]. Previously, we have developed efficient methods to induce differentiation of human iPSCs into alveolar or airway epithelial cells by enriching NKX2-1^+^ lung epithelial progenitor cells [18, 19]. Further, by maintaining and maturing iPSC-derived AT2 cells using SPC-GFP reporter or NaPi2B, an AT2 cell surface antigen, we established human iPSC-based disease models related to abnormalities in lamellar bodies caused by amiodarone, an antiarrhythmic agent notorious for inducing pulmonary fibrosis, and AP3B1 deficiency, which is observed in HPS2 patients [3, 20, 21]. In this study, we generated iPSCs from HPS1 patient-derived fibroblasts with bi-allelic c.1472_1487dup16 variant in *HPS1* gene and their gene-corrected counterparts and differentiated them into lung epithelial cells in organoids to model HPSIP.

## Methods

For details on generation of HPS1 patient-specific iPSCs, teratoma formation assay, karyotyping, immunofluorescence, flow cytometry, western blotting, qRT-PCR, RNA-seq, proteomic analysis and transmission electron microscopy, please refer to Additional file 1: materials and methods.

### Cell culture

All cells were cultured at 37 °C under 5% CO_2_. The culture method for each cell line is described in the respective section.

### Gene correction of HPS1 patient-specific iPSCs

A guide RNA (gRNA), predicted to cause double-strand breaks (DSBs) in the 16-bp duplication in *HPS1* patient-specific genomic DNA sequence and have the most frequent genotype generated by the DSBs accounting for more than 80% of all possible genotypes, was selected using the inDelphi software program [22]. HPS1 patient-specific iPSCs were electroporated to introduce spCas9 and the gRNA expression vector [23]. After single-cell cloning, polymerase chain reaction (PCR) of exon 15 of *HPS1* was performed for screening. Clones with a single 240-bp amplicon were selected and sequenced to confirm the genomic correction of *HPS1* gene. Details on the gene correction method are provided in the supplemental methods.

### Differentiation of iPSCs into NKX2-1^+^ lung epithelial progenitor cells

iPSCs were differentiated into NKX2-1^+^ lung epithelial progenitor cells through the 21-day differentiation method and the progenitor cells were enriched using Carboxypeptidase M (CPM) as their cell surface marker, as detailed in the supplemental methods. Magnetic-activated cell sorting (MACS) or fluorescence-activated cell sorting (FACS) was conducted as described previously [19].

### Generation of CPM-isolated lung bud organoids

CPM-isolated lung bud organoids (C-LBOs) were cultured in the LBO medium containing 3 μM CHIR99021, 10 ng/mL BMP4, 10 ng/mL FGF10, and 50 nM all-trans retinoic acid. Approximately 5 × 10^3^ NKX2-1^+^ lung epithelial progenitor cells were cultured for 7 days in 96 U-bottom plate with low attachment surface to form a spheroid. Next, 2–3 spheroids were embedded in Matrigel in a 24-well cell culture insert and cultured for another 28 days. The culture method of C-LBOs is detailed in the supplemental methods.

### Formation and passage of alveolar organoids

Alveolar organoids (AOs) were cultured in the DCIK medium [20] containing 50 nM dexamethasone, 100 μM 8-Br-cAMP, 100 μM 3-isobutyl-1-methylxanthine, 10 ng/mL KGF. Approximately 1 × 10^4^ NKX2-1^+^ lung epithelial progenitor cells and 5 × 10^5^ precultured human fetal lung fibroblasts (HFLFs) (17.5 weeks of gestation; DV Biologics #PP002-F-1349, lot 121109VA) were embedded in half-diluted Matrigel in a 12-well cell culture insert. NaPi2B^+^ cells were isolated every 14 days and repeatedly cultured in AOs to maintain and mature AT2 cells. AO culture is detailed in the supplemental methods.

### Data availability

All new sequencing data in the present study were deposited in the Gene Expression Omnibus (GEO) under the accession codes GSE179898 and GSE179899.

### Statistical analysis

Data are presented as mean ± SEM. The number of replicates and the statistical tests used are described in each figure legend. Prism7 software program (GraphPad) was used for Student’s t test, Mann–Whitney U test, one-way ANOVA with Tukey’s multiple comparisons test, and two-way ANOVA with Sidak’s multiple comparisons test.

## Results

### Correction of 16-bp microduplication in HPS1 patient-specific iPSCs by genome editing

**Fig. 1:**
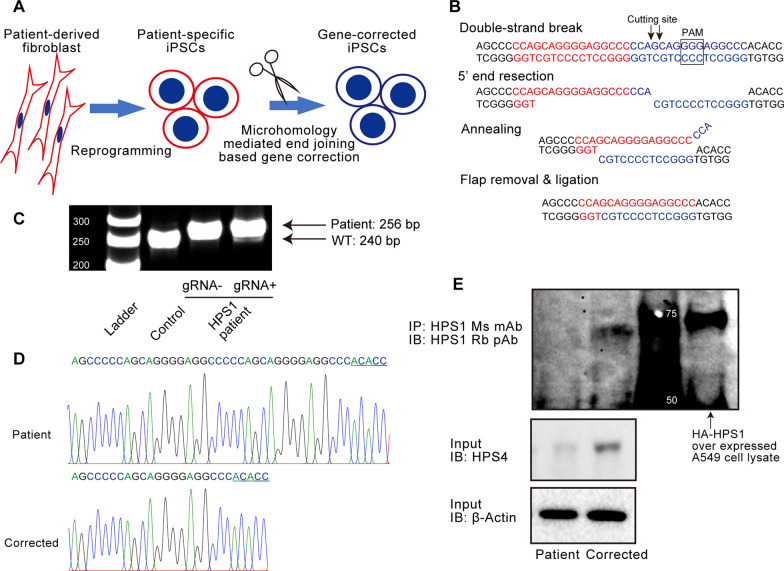
Gene correction of 16-bp microduplication in HPS1 patient-specific iPSCs by microhomology-mediated end joining (MMEJ)-based genome editing. **A** Overview of the generation of patient-specific iPSCs and their gene-corrected counterparts. **B** A presumed MMEJ-based repair mechanism of pathogenic 16-bp microduplication. **C** Genomic PCR of the mutated region in exon 15 of *HPS1*. Control (healthy donor), 240 bp; *HPS1* patient with 16-bp microduplication, 256 bp. Genomic DNA was extracted in bulk from iPSCs transfected with Cas9 and gRNA expression vectors by electroporation. **D** Sequence data of exon 15 in *HPS1* of iPSCs and of those with corrected gene. Gene-corrected iPSCs were selected by a single amplicon of 240 bp after single cell cloning. **E** Verification of gene repair at the protein level by immunoblot. Immunoprecipitation was performed to detect the full-length of HPS1 protein. A549 cell lysate transfected with HA-HPS1 was used as positive control to detect HPS1

HPS1 patient-derived fibroblasts with homozygous 16 bp duplication within exon 15 of the *HPS1* gene (c.1472_1487dup16) were obtained from Coriell Institute for Medical Research (GM14609) and reprogrammed to establish patient-specific iPSCs (Fig. 1A) as described previously [3]. The donor was a 26-year-old woman diagnosed with a possible early stage of pulmonary fibrosis. To confirm the pluripotency of the established iPSCs, a teratoma formation assay was performed, and it revealed that iPSCs differentiated into three germ layers (Additional file 1: Fig. S1A). We decided to use the clone GM14-18 as patient-specific iPSCs for subsequent studies. Next, we generated gene-corrected iPSCs. There are three major mechanisms of gene repair after introducing DNA DSBs: non-homologous end joining (NHEJ), microhomology-mediated end joining (MMEJ), and homologous recombination (HR) [24]. Machine learning models could predict genome editing products from sequences near DSBs in template-free genome editing [22]. In particular, DSBs in pathogenic microduplications efficiently yield a single repair product with a precise mono-sequence through a process mediated by MMEJ. Since the DNA sequence of somatic cells from the same donor could be corrected by template-free genome editing, we used the same approach for gene correction in patient-specific iPSCs [22, 25]. We used the gRNA that could create a DSB in the 16 bp duplication region (Fig. 1B) and transfected it into patient-specific iPSCs with the Cas9 expression vector, as described previously [3]. The PCR product amplified in the genomic sequence near the DSB included a band of approximately the same size as that of control iPSCs (Fig. 1C). After single-cell cloning, 3 out of 92 clones had a single amplicon of approximately the same size as the control iPSCs. All three clones were confirmed to have the homozygotic corrected sequence, which was the same as the one in the healthy donor (Fig. 1D). We decided to use the clone IR9-3 as gene-corrected iPSCs for subsequent studies. Both patient-specific iPSCs (clone GM14-18) and gene-corrected iPSCs (clone IR9-3) expressed undifferentiated markers, and their normal karyotypes were verified (Additional file 1: Fig. S1B–D). The full-length of HPS1 protein is predicted to be 700 amino acids, but c.1472_1487dup16 caused a frameshift with translational termination at codon 586 [26]. When functioning normally, HPS1 protein forms a BLOC-3 complex with HPS4 and stabilizes it [27]. Immunoblotting showed that full-length HPS1 protein was expressed and HPS4 was stabilized in the gene-corrected iPSCs (Fig. 1E).

### Abnormal morphology of HPS1 patient-specific lung bud organoids via TGFβ-IL11 axis

**Fig. 2:**
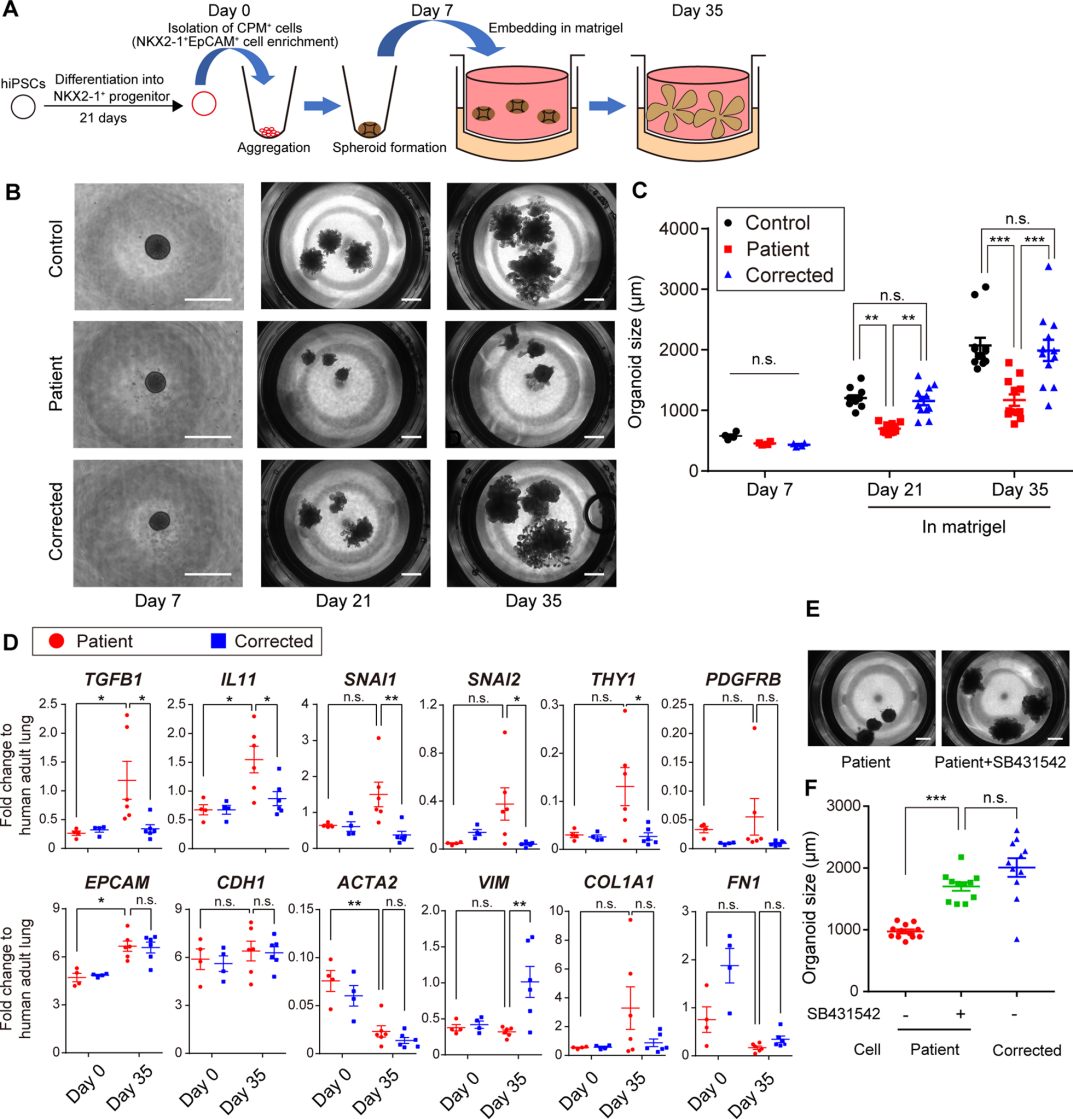
Abnormal morphology of HPS1 patient-specific CPM-isolated lung bud organoids (C-LBOs). **A** Overview of the generation of C-LBOs from iPSCs. **B** Chronological observation of C-LBOs. (Scale bars: 1000 μm) **C** Quantification of organoid size. Data are presented as mean ± SEM (on Day 7, n = 4 organoids from 4 independent experiments; on Day 21 and Day 35, n = 12 organoids from 4 independent experiments). Two-way ANOVA with Sidak’s multiple comparisons test: **P < 0.01, ***P < 0.001. **D** Expression levels of cytokines (*TGFB1* and *IL11*), pan-epithelial cell markers (*EPCAM* and *CDH1*), transcription factors (*SNAI1* and *SNAI2*), and mesenchymal cell markers (cytoskeleton, *ACTA2* and *VIM*; cell surface, *PDGFRB* and *THY1*; extra cellular matrix, *COL1A1* and *FN1*) in C-LBOs evaluated by qRT-PCR, normalized to that of human adult lung. Data are presented as mean ± SEM (n = 6 from 5 independent experiments). Two-way ANOVA with Sidak’s multiple comparisons test: *P < 0.05, **P < 0.01. n.s.: not significance. **E**, **F** Morphologies of HPS1 patient-specific C-LBOs on Day 35, treated with 10 μM SB431542 from Day 0 to Day 35. (Scale bars: 1000 μm) Data are presented as mean ± SEM (n = 11 or 12 organoids from 1 independent experiment). One-way ANOVA with Tukey’s multiple comparisons test: ***P < 0.001. n.s.: not significance

HPS1^−/−^ ESC-derived LBOs have been reported to exhibit abnormal branching structures and fibrotic phenotypes, such as deposition of extracellular matrix (ECM) and increase in mesenchymal cells in three-dimensional culture in Matrigel [17]. Strikoudis et al. speculated that epithelial cell-derived IL11 is a key factor responsible for HPS-related fibrotic phenotypes [28], however, the origin of HPS-related pathogenic mesenchymal cells was unclear due to their differentiation method, in which cells from epithelial and mesenchymal lineages co-differentiated. In our previous study, we proposed an efficient differentiation method for lung epithelial cells by enriching of NKX2-1^+^ progenitor cells using CPM as the surface marker [18]. Here, we developed a new lung organoid model, named C-LBOs, derived only from CPM^+^ epithelial cells to assess whether a lung epithelial cell-only model could recapitulate their HPS-related phenotypes (Fig. 2A). We differentiated and enriched NKX2-1^+^ progenitor cells by isolating the cells expressing CPM on their surface, as described previously [20]. Although EpCAM^−^THY1^+^ or NKX2-1^−^ cells were present before CPM-based isolation (Additional file 1: Fig. S2A, B), EPCAM^+^NKX2-1^+^ epithelial cells were highly enriched after MACS (Additional file 1: Fig. S2C). Consistently, qRT-PCR analysis revealed higher levels of *EPCAM* and *NKX2-1* and lower levels of *VIM* transcripts in the cells after MACS than in the human adult lung tissue (Additional file 1: Fig. S2D), confirming the enrichment of lung epithelial progenitor cells using CPM as the surface antigen. *HPS1* expression in lung progenitor cells from patient-specific iPSCs was remarkably lower than that in gene-corrected iPSC-derived progenitor cells, suggesting the induction of nonsense-mediated mRNA decay (NMD) [29].

The lung progenitor cells derived from each iPSC line were aggregated for 7 days to form spheroids and cultured in Matrigel for another 28 days. In agreement with the previous reports [17, 28], spheroids from healthy donor-derived and *HPS1* gene-corrected iPSCs grew to form a budding structure, while those from the patient-specific iPSCs were smaller and less sharply defined (Fig. 2B, C). Furthermore, the expression of profibrotic cytokines, *TGFB1* and *IL11*, was upregulated in patient-specific C-LBOs on Day 35 (Fig. 2D). The increase was not observed on Day 0, before Matrigel embedding, and was only observed in patient-specific C-LBO cells on Day 35. Although the expression of *SNAI1* and *SNAI2*, transcription factors contributing to epithelial-mesenchymal transition (EMT) [30], increased, the expression of the representative epithelial and mesenchymal cell markers did not differ between patient-derived and gene-corrected iPSCs besides that of *THY1* and *VIM*. HPS4^−/−^ ESC-derived LBOs were reported to show abnormal morphology similar to that of HPS1^−/−^ ESC-derived ones, and the morphology was restored in HPS4^−/−^ IL11^−/−^ ESC-derived LBOs. Since TGFβ was reported to be able to function as an upstream regulator of IL11 [31], we evaluated whether TGFβ signaling could be involved in the epithelial abnormalities of HPS1 patient-specific C-LBOs. Addition of SB431542, an inhibitor of TGFβ signaling, corrected the morphological abnormalities of HPS1 patient-specific C-LBOs (Fig. 2E, F). We then generated HPS1 knockout (KO) A549 cells to validate the epithelial cell phenotypes induced by HPS1 deficiency (Additional file 1: Fig. S3A–C). *IL11*, *TGFB1*, *SNAI1*, *SNAI2,* and *THY1* were upregulated in HPS1 KO A549 cells, consistent with the findings in HPS1 patient-specific C-LBOs, and we observed that the upregulation was suppressed by SB431542 (Additional file 1: Fig. S3D). These results suggested that C-LBO and A549 cell phenotype caused by HPS1 deficiency was mediated by the TGFβ-IL11 axis.

Next, we evaluated the cell lineages contained in the organoids using a healthy donor-derived iPSCs. Almost all cells in healthy donor-derived C-LBOs expressed EpCAM on Day 35 (Additional file 1: Fig. S4A). However, NKX2-1, an essential transcription factor for the differentiation into lung epithelial cells, was remarkably downregulated (Additional file 1: Fig. S4B, C). Furthermore, SOX2, a transcription factor involved in proximal lung development [32], was decreased, although SOX9, a transcription factor involved in distal lung development [33], was upregulated. Airway epithelial cell markers were not significantly upregulated on Day 35 compared with that in progenitor cells, but p63^+^ cells were occasionally present (Additional file 1: Fig. S4D, E). In addition, there was little expression of AT2 cell markers compared with that in adult lungs (Additional file 1: Fig. S4F). Although our C-LBOs might reflect some pathological change similar to that in HPSIP, failure to show the robust presence of AT2 cells pushed us to simulate lung phenotype of HPS1 in a model where AT2 cells are present.

### Profiling of HPS1 patient-specific AOs

**Fig. 3:**
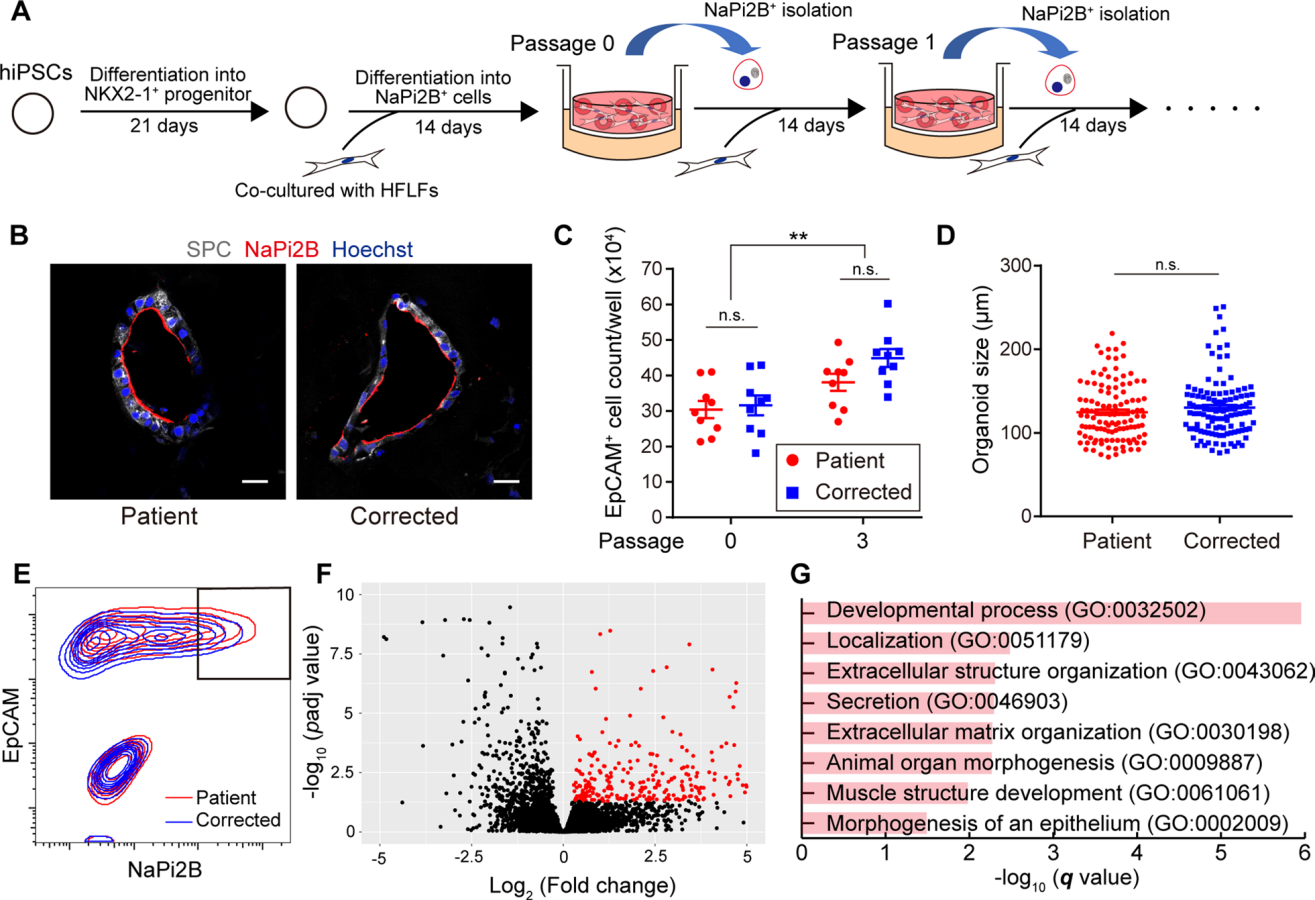
Profiling of HPS1 patient-specific alveolar organoids (AOs). **A** Overview of the generation of AOs from iPSCs. **B** Immunofluorescent imaging of AOs. Gray, SPC; Red, NaPi2B; Blue, nuclei (Hoechst). (Scale bars: 20 μm) **C** Total number of EpCAM^+^ cells in a well. The manually counted total number of dissociated cells was multiplied by the ratio of EpCAM^+^ cells obtained from flow cytometric analysis. Data are presented as mean ± SEM (n = 9 from 9 independent experiments). Two-way ANOVA with Sidak’s multiple comparisons test: **P < 0.01. n.s.: not significance. **D** Quantification of organoid size. Data are presented as mean ± SEM (n = 120 organoids from 6 independent experiments). Mann–Whitney U test. **E** Gating on flow cytometry for transcriptomic analysis of NaPi2B^high^ cells. Representative flow cytometry plots of HPS1 patient-specific AOs and their gene-corrected counterparts are shown. AOs after 3 passages using NaPi2B-based sorting were used for analysis. **F** Volcano plot of differentially expressed genes (DEGs) of NaPi2B^high^ cells in HPS1 patient-specific AOs compared with the gene-corrected counterparts. The volcano plot shows log_2_ (fold change) and −log_10_ (*p*adj value) calculated by DESeq2. Significantly upregulated 302 genes with *p*adj value < 0.05 and log_2_ (fold change) > 0 are plotted in red. **G** Gene ontology enrichment analysis of biological process using the upregulated 302 genes in NaPi2B^high^ cells of HPS1 patient-specific AOs over the gene-corrected counterparts

For profiling of AT2 cells derived from HPS1 patient-specific iPSCs, NKX2-1^+^ lung progenitor cells were co-cultured with human fetal lung fibroblasts (HFLFs) embedded in Matrigel to form AOs (Fig. 3A), as described previously [3, 20]. The resulting iPSC-derived AOs expressed various AT2 cell markers and could be passaged using NaPi2B expression as an AT2 cell surface marker (Fig. 3B and Additional file 1: Fig. S5A–C). In contrast to C-LBO model, there was no significant difference in the total number of epithelial cells or in the size of each organoid between patient-specific and gene-corrected AOs (Fig. 3C, D). In transcriptomic analysis of isolated NaPi2B^high^ cells, which enriched AT2 cells, 302 genes were significantly upregulated in patient-specific AOs (Fig. 3E, F, and Additional file 2: Table S5). Gene ontology (GO) enrichment analysis of these genes showed that “localization” and “secretion” related genes were enriched (Fig. 3G). These results were justified since HPS1 is a protein associated with membrane trafficking. Further, the enriched genes annotated to “development process”, “animal organ morphogenesis”, and “morphogenesis of an epithelium” suggested that deficiency of HPS1 could affect development of lung epithelial cells in vitro.

### Different transcriptomes between AOs and LBOs in HPS1-disease models

**Fig. 4:**
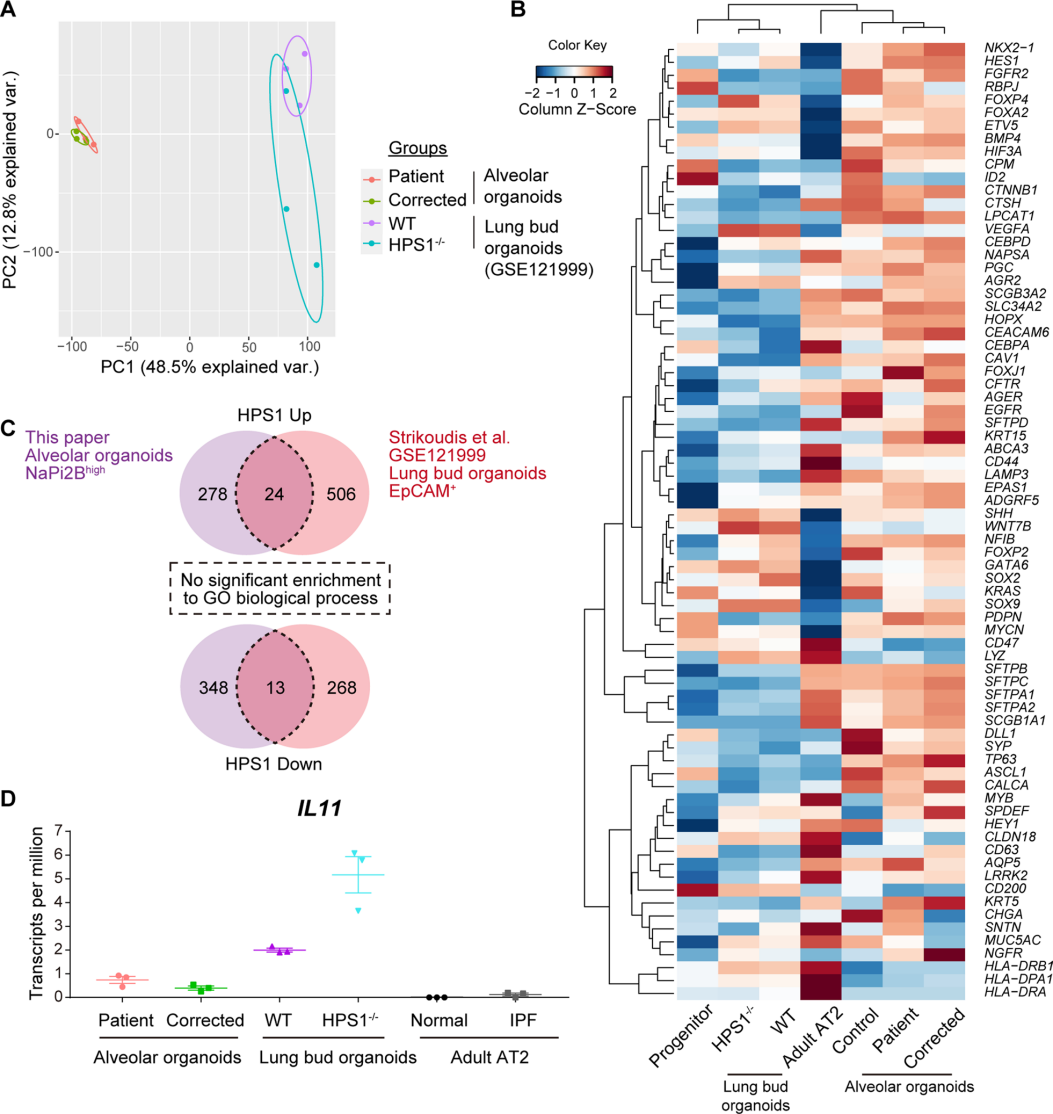
Transcriptomic comparison between alveolar organoids (AOs) and lung bud organoids (LBOs) in HPS1-disease models. **A** Principal component analysis (PCA) of the transcriptomes of AOs and LBOs in HPS1-disease models. Log_2_ (TPM + 0.01) (transcripts per million) were used for PCA. **B** Hierarchical clustering based on the expression of lung epithelial cell markers. A heatmap indicating Z-score of each gene. Mean values of log_2_ (TPM + 0.01) were used to calculate the Z-score. The following data sets were used: CPM^high^ progenitor cells, GES163575; Lung bud organoids, GSE121999; Adult AT2 cells, GSE131768; Alveolar organoids, GSE179898 and GSE179899. **C** Overlapped upregulated or downregulated DEGs on each model and their results of gene ontology enrichment analysis of biological process. **D** TPM of *IL11* on each model. The following data sets were used: adult AT2 cells with normal and IPF conditions, GSE94555

We used transcriptome analysis to compare the expression of epithelial cell lineage markers of AOs and LBOs in HPS1-disease models. Since we could not find a robust expression of AT2 cell markers in our C-LBOs, we hypothesized that our methodological differences such as surface marker-based sorting of lung progenitor cells might have caused low levels of AT2 cell markers in the organoids. Then, we used the published transcriptomic data of LBOs (GSE121999; Additional file 3: Table S6), which matched the transcriptome of second-trimester human fetal lung [28]. In principal component analysis (PCA) of whole transcriptome, AOs and LBOs were separated in different clusters (Fig. 4A). A hierarchical clustering analysis based on lung epithelial cell markers showed that AOs showed greater similarity to primary adult AT2 cells [34] than LBOs. The LBOs showed lower expression of AT2 markers (*SFTPA1*, *SFTPA2*, *SFTPB*, *SFTPC*, *SFTPD*, *ABCA3*, *LAMP3*, *NAPSA*) than did primary adult AT2 cells and clustered in the same group of our CPM^+^ lung progenitor cells (Fig. 4B and Additional file 4: Table S7) [20]. Differentially expressed genes (DEGs) induced by HPS1 deficiency in each model did not overlap as much (Fig. 4C). Overlapped genes revealed no significant enrichment of GO biological processes. We found that finding common phenotypes of HPS1 deficiency by comparing transcriptomes of each model of AOs and LBOs was difficult. Strikingly, the expression of *IL11* in AOs was lower than that in LBOs and was not substantially upregulated in patient-specific AOs as in gene-corrected AOs (Fig. 4D). Furthermore, the expression of *TGFB1* was not upregulated in patient-specific AOs compared with that in gene-corrected AOs (Additional file 2: Table S5). Although *IL11* is upregulated in the IPF whole lung [35], it was not upregulated in HTII-280 positive cells of IPF lung (Fig. 4D) [36]. To identify *IL11*-expressing cells, single-cell RNA-seq data were analyzed [37, 38]. Although *IL11* expression was low in most lung cell types, some cells classified as “Aberrant Basaloid” and “Alveolar fibroblast” expressed *IL11* in IPF (Additional file 1: Fig. S6). Clinical observation and analysis of mouse model have shown that AT2 cells play critical roles in interstitial pneumonia, including HPSIP [39]. Transcriptome analysis of AOs and LBOs showed that AOs could contain more AT2 cells and could mimic the alveolar epithelium better than LBOs could; therefore, we decided to use AOs for disease modeling.

### Disease modeling of patient-specific iPSCs derived AOs

**Fig. 5:**
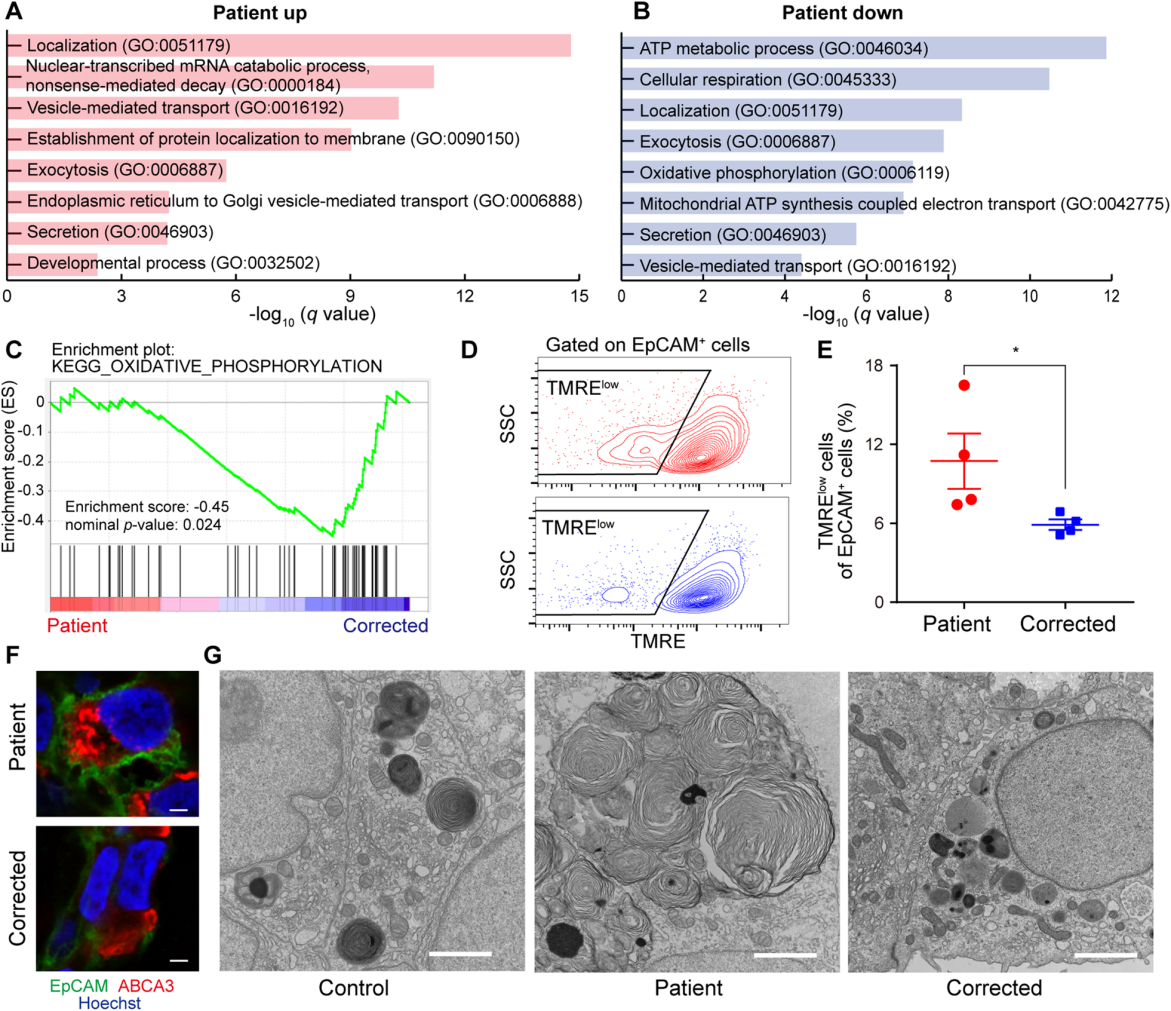
Disease modeling of HPS1 in the patient-derived AOs. **A**, **B** Proteomic analysis of EpCAM^+^ cells isolated from AOs. EpCAM^+^ cells in HPS1 patient-specific and their gene-corrected AOs after 2 passages using NaPi2B-based sorting were subjected to further analysis. Gene ontology enrichment analysis of biological process was performed for upregulated proteins with log_2_ (fold change) > 0.5 or downregulated proteins with log_2_ (fold change) < − 0.5. **C** Gene set enrichment analysis to “KEGG_OXIDATIVE_PHOSPHORYLATION” for comparison of protein expression between HPS1 patient-specific iPSC-derived epithelial cells of AOs with their gene-corrected counterparts. **D**, **E** Flow cytometric analysis of mitochondrial membrane potential using tetramethylrhodamine, ethyl ester (TMRE). AOs after 2–4 passages using NaPi2B-based sorting were used for analysis. Upper dot plot: patient-derived EpCAM^+^ cells. Lower dot plot: gene-corrected counterparts. Data are presented as mean ± SEM (n = 4 from 3 independent experiments). Mann–Whitney U test: *P < 0.05. **F** Immunofluorescent imaging of AOs in each condition. Green, EpCAM; Red, ABCA3; Blue, nuclei (Hoechst). (Scale bars: 2 μm) **G** Transmission electron microscope images of lamellar bodies in AOs in each condition. (Scale bars: 2 μm)

We performed proteomic analysis to search for disease phenotypes in HPS1 patient-specific AOs at the protein level (Additional file 5: Table S8). GO enrichment analysis of upregulated proteins in EpCAM^+^ cells isolated from the patient-specific AOs showed that several enriched GO biological processes were consistent with transcriptome analysis, such as “localization”, “secretion”, and, “developmental process” (Fig. 5A). On the other hand, GO terms remarkable in transcriptomic analysis, such as “extracellular matrix organization” and “muscle contraction” were not enriched in the proteomic analysis. GO enrichment analysis of downregulated proteins in EpCAM^+^ cells isolated from the patient-specific AOs showed the GO terms common to those upregulated proteins, such as “localization”, “exocytosis”, “secretion”, and “vesicle-mediated transport” (Fig. 5B). However, surprisingly, mitochondrial function related to GO terms, such as “ATP metabolic process”, “cellular respiration”, “oxidative phosphorylation” and “Mitochondrial ATP synthesis coupled electron transport” were detected only in the GO enrichment analysis of downregulated proteins. Furthermore, gene set enrichment analysis (GSEA) supported the hypothesis that mitochondrial function, such as oxidative phosphorylation (OXPHOS), was declined in the patient-specific alveolar epithelial cells (Fig. 5C). Mitochondrial membrane potential associated with OXPHOS drives the ATP synthase to supply the majority of ATP in eukaryotic cells. Hence evaluation of mitochondrial membrane potential is one of the standard methods for analysis of mitochondrial function [40]. The number of EpCAM^+^ cells with reduced mitochondrial membrane potential was slightly increased in patient-specific AOs (Fig. 5D, E). Intracellular reactive oxygen species (ROS) are mainly generated in the mitochondria, and mitochondrial ROS levels were reported to be increased in the lung tissue of IPF and alveolar epithelial cells of HPS2 model mice [41, 42]. Consistent with these reports, the accumulation of intracellular ROS was observed in HPS1 KO A549 cells (Additional file 1: Fig. S7A–C). These results suggested that deficiency of HPS1 in alveolar epithelial cells impaired mitochondrial function.

AT2 cells have lamellar bodies storing pulmonary surfactant lipids and proteins. These lamellar bodies are formed as LROs from the Golgi apparatus and their lamellar structure is composed of phospholipids that are transported by ABCA3 present in the limiting membrane [43]. Giant lamellar bodies in AT2 cells were reported to be the salient histopathologic feature of HPSIP [11], probably caused by impaired membrane trafficking in HPS. We confirmed the expression of ABCA3 in the AOs (Fig. 5F) and observed the morphology of lamellar bodies in transmission electron microscopy. In the patient-specific AOs, we observed the formation of lamellar bodies of different morphologies and sizes (Additional file 1: Fig. S8A, B). Some of them showed remarkably enlarged morphology similar to giant lamellar bodies reported in the HPS1 patient’s lung tissue (Fig. 5G). On the other hand, no giant lamellar body-like structure was observed in the healthy donor-derived control or gene-corrected AOs.

## Discussion

While tissue-derived somatic cells from rare disease patients are generally difficult to obtain and culture, patient-specific iPSCs can overcome this hurdle. We established HPS1 patient-specific iPSCs with the major bi-allelic 16-bp duplication mutation and their gene-corrected iPSCs to study HPSIP in two different lung organoid models: C-LBOs and AOs. C-LBOs showed abnormal morphology and increased *IL11* expression. AOs comprising iPSC-derived AT2 cells and primary pulmonary fibroblasts showed mitochondrial dysfunction in epithelial cells and exhibited “giant lamellar body”—a salient histopathologic feature of HPSIP (Additional file 1: Table S9).

Our C-LBOs derived from CPM^+^ lung epithelial progenitors with more than 80% NKX2-1^+^ cells did not show consistent lung epithelial cell marker expression and were not able to identify typical cell lineages. Only distal tip cells at the edge of the C-LBOs maintained NKX2-1, while 75% of the entire cells expressed NKX2-1 in the LBOs in the previous report [17]. This difference may be explained by either the presence or absence of mesenchymal cells, or by organoid construction methods: forced aggregation in our method and selection of the organoids with spontaneous folding structures in the other method [44]. Our forced aggregation method produced uniform organoids, which allowed us to quantify the size of the organoids, but induction of the specific lineage of lung epithelial cells, such as AT2 cells, remains a challenge. *TP63* and *SOX9* were expressed in healthy donor-derived control iPSC-derived C-LBOs at equal or higher levels than in adult human lungs, whereas *KRT5* expression was very low. This may partly mimic some features of aberrant basaloid cells, whose number is increased in the IPF distal lung [38]. In HPS1 patient-specific iPSC-derived C-LBOs, upregulation of *TGFB1*, *IL11* and some EMT-related transcription factors was observed in addition to morphological abnormality. Consistently, aberrant basaloid cells exhibit EMT features, and our reanalysis of single cell RNA-seq data showed that they could be a source of *IL11*. In fact, IL11 has been reported to promote fibroblast activation and cause EMT in epithelial cells [35, 45]. This EMT-like phenotype, even though partial, could promote a pro-fibrotic microenvironment in autocrine and paracrine manner [46]. Jonsdottir et al. reported that a layer of p63^+^ epithelial cells overlying fibroblastic foci in IPF showed partial EMT, in which they expressed both epithelial and mesenchymal cell markers [47]. They also reported that a human bronchial-derived basal epithelial cell line grew to show branching structures; however, the cell line with an EMT inducer did not show such structures [47]. Based on these findings, the phenotype of our HPS1 patient-specific iPSC-derived C-LBOs might have recapitulated a partial EMT of basal-like cells seen in IPF patients. These results suggest a new function of HPS1. However, whether it is possible to detect abnormalities caused by HPS1-deficiency in cells other than alveolar epithelial cells warrants further research.

In the analysis of AOs, which include AT2 cells with LROs, there were no significant differences in organoid morphology and size between HPS1 patient-specific and gene-corrected iPSC-derived AOs. Furthermore, upregulated gene expression of isolated NaPi2B^high^ cells in patient-specific AOs did not show enrichment of EMT-related genes, such as *SNAI1* and *SNAI2*. There are two possible interpretations of phenotype with no EMT in our AOs. First, the need for periodic passaging with NaPi2B expression might exclude the EMT-prone cell population, and second, the co-culture with normal fibroblasts might make it difficult to distinguish between EMT-prone epithelial cells and native fibroblasts. Although our AOs have these limitations for proving that AT2 cells in HPS1 patient did not show EMT-like phenotype, lineage tracing of bleomycin mouse model and histochemical analysis of HPS mouse model supported our observations for two organoid models [48, 49]. Proteomic analysis revealed that HPS1 patient-specific AOs could show impaired membrane trafficking and mitochondrial dysfunction. Abnormalities in membrane trafficking were observed in several hereditary interstitial lung diseases, such as SFTPC with I73T mutation and HPS, and mitochondrial dysfunction in AT2 cells was reported as a common phenotype [42, 50–52]. Our results could reinforce the possibility of a relationship between abnormal membrane trafficking and mitochondrial dysfunction. Although we were able to recapitulate the giant lamellar body in HPS1 deficiency in vitro, their frequency and size varied greatly. Therefore, they could not be used directly to develop quantitative assays for drug discovery. Furthermore, whether HPS1 patient-specific AOs can recapitulate fibroblast activation induced by epithelial cell dysfunction, which is considered as an important step in the pathogenesis of pulmonary fibrosis [53], has not yet been demonstrated. Finally, since the immaturity of iPSC-derived AT2 cells in AOs [20] might interrupt the modeling of late-onset disease, including HPSIP, we need to search for conditions that can accelerate the disease progression or maturate the iPSC-derived cells in the future.

## Conclusions

This study provides new research approaches toward understanding the pathogenesis of HPSIP caused by HPS1 deficiency.

## Supplementary Information

**Additional file 1**: Additional material and methods, **Fig. S1**. Generation of HPS1 patient-specific iPSCs and their gene-corrected counterparts, related to Fig. 1. (A) Teratoma assay of HPS1 patient-specific iPSCs for evaluating pluripotency. Representative images of hematoxylin and eosin staining: ectoderm, neuronal cells; endoderm, gland lumen; mesoderm, cartilage. (Scale bars: 100 μm) (B) Immunofluorescent imaging of representative undifferentiation markers (NANOG, SOX2, OCT3/4, SSEA-4). (Scale bar: 50 μm) (C) TRA1-60 positive cell rate using flow cytometric analysis. (D) Karyotypes of HPS1 patient-specific iPSCs (clone GM14-18) and their gene-corrected counter parts (clone IR9-3). **Fig. S2.** Isolation of NKX2-1^+^EpCAM^+^ cells using CPM as a surface antigen, related to Fig. 2. (A) Flow cytometric analysis of EpCAM, THY1 and CPM in a control cell line after 21-day stepwise differentiation. (B) Flow cytometric analysis of NKX2-1 and CPM in each cell line. (C) Histogram of EpCAM^+^ and NKX2-1^+^ cells sorted based on the expression of CPM using MACS. (D) Expression of marker genes in isolated lung progenitor cells of each cell line evaluated by qRT-PCR normalized to that of human adult lung. Data are presented as mean ± SEM (n = 4 from 4 independent experiments). **Fig. S3.** Validation of phenotypes observed in patient-specific C-LBOs using HPS1KO A549 cells, related to Fig. 2. (A) Sequence data of wild type (WT) and HPS1 knockout (KO) A549 cells. (B) Verification of HPS1 KO at the protein level by immunoblot. Immunoprecipitation was performed to detect the full-length of HPS1 protein. A549 cell lysate transfected with HA-HPS1 was used as a positive control to detect HPS1. (C) Nonsense mediated decay of HPS1 mRNA in HPS1KO A549 cells determined by qRT-PCR, normalized to that of human adult lung. Data are presented as mean ± SEM (n = 3 from 3 independent experiments). Unpaired two-tailed Student’s t test: ***P < 0.001. (D) Expression levels of cytokines (*IL11* and *TGFB1*), transcription factors (*SNAI1* and *SNAI2*), and a mesenchymal cell marker (*THY1*) in HPS1 KO A549 cells, treated with 10 μM SB431542 for 3 days determined by qRT-PCR, normalized to that of human adult lung. Data are presented as mean ± SEM (n = 6 from 3 independent experiments). One-way ANOVA with Tukey’s multiple comparisons test: **P < 0.01, ***P < 0.001. **Fig. S4.** Analysis of cell lineages in CPM-isolated lung bud organoids derived from healthy-donor derived control iPSCs on Day 35, related to Fig. 2. (A) Ratio of EpCAM^+^ cells on Day 35 by flow cytometric analysis. HFLFs were used as a negative control. (B) Gene expression of the pan-epithelial (*EPCAM*), pan-mesenchymal (*VIM*), and transcription factors related to lung development (*NKX2-1*, *SOX2*, and *SOX9*) determined by qRT-PCR, normalized to that of human adult lung (n = 3 from 3 independent experiments). (C) Immunofluorescent images on Day 35 (Scale bars: 500 μm, C). (D) Gene expression of airway epithelial cell markers determined by qRT-PCR normalized to that of human adult lung. (E) Immunofluorescent imaging on Day 35. Red, p63; Gray, EpCAM; Blue, nuclei (Hoechst). (Scale bars: 50 μm) (F) Gene expression of AT2 cell markers by qRT-PCR normalized to that of human adult lung. **Fig. S5.** Passage of alveolar organoids (AOs) using NaPi2B as a surface antigen, related to Fig. 3. (A) Gating on flow cytometry for passaging AOs. Representative flow cytometry plots at passage 0 of HPS1 iPSC-derived AOs and their gene-corrected counterparts are shown. (B) Ratio of NaPi2B^+^ cells in EpCAM^+^ cells during the periodic passaging of AOs. Data are presented as mean ± SEM (n = 3 from 3 independent experiments). (C) AT2 marker gene expression during the periodic passaging of AOs determined by qRT-PCR, normalized to that of human adult lung. Data are presented as mean ± SEM (n = 3 from 3 independent experiments). **Fig. S6.** Analysis of *IL11*-expressing cells using the IPF cell atlas, related to Fig. 4. We used Kaminski/Rosas data set (www.ipfcellatlas.com). **Fig. S7.** Validation of phenotypes observed in patient-specific AOs using HPS1 KO A549 cells, related to Fig. 5. (A) Live cell imaging of intracellular reactive oxygen species (ROS) of HPS1 KO A549 cells using DCFH-DA dye and MitoTracker Deep Red FM. (Scale bars: 20 μm). (B and C) Quantification of DCFH-DA staining intensity in HPS1 KO A549 cells by flow cytometry. Data are presented as mean ± SEM (n = 3 from 3 independent experiments). Unpaired two-tailed Student’s t test: *P < 0.05. MFI: Mean Fluorescence Intensity. **Fig. S8.** Heterogeneous lamellar bodies of HPS1 patient-specific alveolar organoids (AOs), related to Fig. 5. Transmission electron microscope images of lamellar bodies in patient-specific AOs (Scale bar: 5 μm). (A) Both abnormal and normal-shaped lamellar bodies were present in HPS1 patient-specific alveolar epithelial cells in organoids. (B) Miscellaneous lamellar body structures in HPS1 patient-specific AOs: left, normal; center, sparsely expanded lamellar structure but not spherical; right, excessively fused lamellar bodies. **Table S1.** Primary antibodies used in the present study. **Table S2.** Secondary antibodies used in the present study. **Table S3.** Primers for TaqMan qPT-PCR.** Table S4.** Primers for SYBR green qRT-PCR.** Table S9.** Summary of the organoid model.

**Additional file 2: Table S5.** RNA-seq processed data of NaPi2B^high^ cells isolated from alveolar organoids.

**Additional file 3: Table S6.** RNA-seq processed data of EpCAM^+^ cells isolated from lung bud organoids, reanalysis of the publicly available data (GSE121999).

**Additional file 4: Table S7.** RNA-seq processed data of each model on lung epithelial cell marker gene expression.

**Additional file 5: Table S8**. Proteomic data of EpCAM^+^ cells isolated from alveolar organoids.

## Data Availability

All new sequencing data in the present study were deposited in the Gene Expression Omnibus (GEO) under the accession codes GSE179898 and GSE179899.

## Abbreviations

iPSCs: Induced pluripotent stem cells
HPS: Hermansky–Pudlak syndrome
HPSIP: HPS associated interstitial pneumonia
AT2: Alveolar type 2
ESCs: Embryonic stem cells
LROs: Lysosome-related organelles
IPF: Idiopathic pulmonary fibrosis
LBOs: Lung bud organoids
PCR: Polymerase chain reaction
CPM: Carboxypeptidase M
MACS: Magnetic-activated cell sorting
FACS: Fluorescence-activated cell sorting
C-LBOs: CPM-isolated lung bud organoids
AOs: Alveolar organoids
DSB: Double-strand breaks
NHEJ: Non-homologous end joining
MMEJ: Microhomology-mediated end joining
HR: Homologous recombination
ECM: Extracellular matrix
NMD: Nonsense-mediated mRNA decay
EMT: Epithelial-mesenchymal transition
KO: Knock out
HFLFs: Human fetal lung fibroblasts
PCA: Principal component analysis
DEGs: Differentially expressed genes
GSEA: Gene set enrichment analysis
OXPHOS: Oxidative phosphorylation
ROS: Reactive oxygen species
TMRE: Tetramethylrhodamine, ethyl ester

## References

[1] Liu G, David BT, Trawczynski M, Fessler RG (2020). Advances in pluripotent stem cells: history, mechanisms, technologies, and applications. Stem Cell Rev Rep.

[2] Shi Y, Inoue H, Wu JC, Yamanaka S (2017). Induced pluripotent stem cell technology: a decade of progress. Nat Rev Drug Discov.

[3] Korogi Y, Gotoh S, Ikeo S, Yamamoto Y, Sone N, Tamai K, Konishi S, Nagasaki T, Matsumoto H, Ito I (2019). In vitro disease modeling of Hermansky–Pudlak syndrome type 2 using human induced pluripotent stem cell-derived alveolar organoids. Stem Cell Rep.

[4] Paik DT, Chandy M, Wu JC (2020). Patient and disease-specific induced pluripotent stem cells for discovery of personalized cardiovascular drugs and therapeutics. Pharmacol Rev.

[5] Huizing M, Malicdan MCV, Gochuico BR, Gahl WA. Hermansky–Pudlak Syndrome. In: Adam MP, Ardinger HH, Pagon RA, Wallace SE, Bean LJH, Mirzaa G, Amemiya A, editors. GeneReviews. University of Washington, Seattle. 2000 (updated 2021 Mar 18). 20301464

[6] Bowman SL, Bi-Karchin J, Le L, Marks MS (2019). The road to lysosome-related organelles: insights from Hermansky–Pudlak syndrome and other rare diseases. Traffic.

[7] Vicary GW, Vergne Y, Santiago-Cornier A, Young LR, Roman J (2016). Pulmonary fibrosis in Hermansky–Pudlak syndrome. Ann Am Thorac Soc.

[8] Velázquez-Díaz P, Nakajima E, Sorkhdini P, Hernandez-Gutierrez A, Eberle A, Yang D, Zhou Y (2021). Hermansky-Pudlak syndrome and lung disease: pathogenesis and therapeutics. Front Pharmacol.

[9] Santiago Borrero PJ, Rodríguez-Pérez Y, Renta JY, Izquierdo NJ, Del Fierro L, Muñoz D, Molina NL, Ramírez S, Pagán-Mercado G, Ortíz I (2006). Genetic testing for oculocutaneous albinism type 1 and 2 and Hermansky–Pudlak syndrome type 1 and 3 mutations in Puerto Rico. J Invest Dermatol.

[10] Yokoyama T, Gochuico BR (2021). Hermansky–Pudlak syndrome pulmonary fibrosis: a rare inherited interstitial lung disease. Eur Respir Rev.

[11] Nakatani Y, Nakamura N, Sano J, Inayama Y, Kawano N, Yamanaka S, Miyagi Y, Nagashima Y, Ohbayashi C, Mizushima M (2000). Interstitial pneumonia in Hermansky–Pudlak syndrome: significance of florid foamy swelling/degeneration (giant lamellar body degeneration) of type-2 pneumocytes. Virchows Arch.

[12] Rouhani FN, Brantly ML, Markello TC, Helip-Wooley A, O'Brien K, Hess R, Huizing M, Gahl WA, Gochuico BR (2009). Alveolar macrophage dysregulation in Hermansky–Pudlak syndrome type 1. Am J Respir Crit Care Med.

[13] Cullinane AR, Yeager C, Dorward H, Carmona-Rivera C, Wu HP, Moss J, O'Brien KJ, Nathan SD, Meyer KC, Rosas IO (2014). Dysregulation of galectin-3. Implications for Hermansky–Pudlak syndrome pulmonary fibrosis. Am J Respir Cell Mol Biol.

[14] Kirshenbaum AS, Cruse G, Desai A, Bandara G, Leerkes M, Lee CC, Fischer ER, O'Brien KJ, Gochuico BR, Stone K (2016). Immunophenotypic and ultrastructural analysis of mast cells in Hermansky–Pudlak syndrome type-1: a possible connection to pulmonary fibrosis. PLoS ONE.

[15] Mahavadi P, Korfei M, Henneke I, Liebisch G, Schmitz G, Gochuico BR, Markart P, Bellusci S, Seeger W, Ruppert C, Guenther A (2010). Epithelial stress and apoptosis underlie Hermansky–Pudlak syndrome-associated interstitial pneumonia. Am J Respir Crit Care Med.

[16] Young LR, Gulleman PM, Bridges JP, Weaver TE, Deutsch GH, Blackwell TS, McCormack FX (2012). The alveolar epithelium determines susceptibility to lung fibrosis in Hermansky–Pudlak syndrome. Am J Respir Crit Care Med.

[17] Chen YW, Huang SX, de Carvalho A, Ho SH, Islam MN, Volpi S, Notarangelo LD, Ciancanelli M, Casanova JL, Bhattacharya J (2017). A three-dimensional model of human lung development and disease from pluripotent stem cells. Nat Cell Biol.

[18] Gotoh S, Ito I, Nagasaki T, Yamamoto Y, Konishi S, Korogi Y, Matsumoto H, Muro S, Hirai T, Funato M (2014). Generation of alveolar epithelial spheroids via isolated progenitor cells from human pluripotent stem cells. Stem Cell Rep.

[19] Konishi S, Gotoh S, Tateishi K, Yamamoto Y, Korogi Y, Nagasaki T, Matsumoto H, Muro S, Hirai T, Ito I (2016). directed induction of functional multi-ciliated cells in proximal airway epithelial spheroids from human pluripotent stem cells. Stem Cell Rep.

[20] Yamamoto Y, Gotoh S, Korogi Y, Seki M, Konishi S, Ikeo S, Sone N, Nagasaki T, Matsumoto H, Muro S (2017). Long-term expansion of alveolar stem cells derived from human iPS cells in organoids. Nat Methods.

[21] Kanagaki S, Suezawa T, Moriguchi K, Nakao K, Toyomoto M, Yamamoto Y, Murakami K, Hagiwara M, Gotoh S (2021). Hydroxypropyl cyclodextrin improves amiodarone-induced aberrant lipid homeostasis of alveolar cells. Am J Respir Cell Mol Biol.

[22] Shen MW, Arbab M, Hsu JY, Worstell D, Culbertson SJ, Krabbe O, Cassa CA, Liu DR, Gifford DK, Sherwood RI (2018). Predictable and precise template-free CRISPR editing of pathogenic variants. Nature.

[23] Li HL, Fujimoto N, Sasakawa N, Shirai S, Ohkame T, Sakuma T, Tanaka M, Amano N, Watanabe A, Sakurai H (2015). Precise correction of the dystrophin gene in duchenne muscular dystrophy patient induced pluripotent stem cells by TALEN and CRISPR-Cas9. Stem Cell Rep.

[24] Jang HK, Song B, Hwang GH, Bae S (2020). Current trends in gene recovery mediated by the CRISPR-Cas system. Exp Mol Med.

[25] Iyer S, Suresh S, Guo D, Daman K, Chen JCJ, Liu P, Zieger M, Luk K, Roscoe BP, Mueller C (2019). Precise therapeutic gene correction by a simple nuclease-induced double-stranded break. Nature.

[26] Oh J, Bailin T, Fukai K, Feng GH, Ho L, Mao JI, Frenk E, Tamura N, Spritz RA (1996). Positional cloning of a gene for Hermansky–Pudlak syndrome, a disorder of cytoplasmic organelles. Nat Genet.

[27] Ikawa Y, Hess R, Dorward H, Cullinane AR, Huizing M, Gochuico BR, Gahl WA, Candotti F (2015). In vitro functional correction of Hermansky–Pudlak Syndrome type-1 by lentiviral-mediated gene transfer. Mol Genet Metab.

[28] Strikoudis A, Cieślak A, Loffredo L, Chen YW, Patel N, Saqi A, Lederer DJ, Snoeck HW (2019). Modeling of fibrotic lung disease using 3D organoids derived from human pluripotent stem cells. Cell Rep.

[29] Hazelwood S, Shotelersuk V, Wildenberg SC, Chen D, Iwata F, Kaiser-Kupfer MI, White JG, King RA, Gahl WA (1997). Evidence for locus heterogeneity in Puerto Ricans with Hermansky–Pudlak syndrome. Am J Hum Genet.

[30] Zeisberg M, Neilson EG (2009). Biomarkers for epithelial-mesenchymal transitions. J Clin Invest.

[31] Kortekaas RK, Burgess JK, van Orsoy R, Lamb D, Webster M, Gosens R (2021). Therapeutic targeting of IL-11 for chronic lung disease. Trends Pharmacol Sci.

[32] Gontan C, de Munck A, Vermeij M, Grosveld F, Tibboel D, Rottier R (2008). Sox2 is important for two crucial processes in lung development: branching morphogenesis and epithelial cell differentiation. Dev Biol.

[33] Rockich BE, Hrycaj SM, Shih HP, Nagy MS, Ferguson MA, Kopp JL, Sander M, Wellik DM, Spence JR (2013). Sox9 plays multiple roles in the lung epithelium during branching morphogenesis. Proc Natl Acad Sci U S A.

[34] Hurley K, Ding J, Villacorta-Martin C, Herriges MJ, Jacob A, Vedaie M, Alysandratos KD, Sun YL, Lin C, Werder RB (2020). Reconstructed single-cell fate trajectories define lineage plasticity windows during differentiation of human PSC-derived distal lung progenitors. Cell Stem Cell.

[35] Ng B, Dong J, D'Agostino G, Viswanathan S, Widjaja AA, Lim WW, Ko NSJ, Tan J, Chothani SP, Huang B (2019). Interleukin-11 is a therapeutic target in idiopathic pulmonary fibrosis. Sci Transl Med.

[36] Xu Y, Mizuno T, Sridharan A, Du Y, Guo M, Tang J, Wikenheiser-Brokamp KA, Perl AT, Funari VA, Gokey JJ (2016). Single-cell RNA sequencing identifies diverse roles of epithelial cells in idiopathic pulmonary fibrosis. JCI Insight.

[37] Neumark N, Cosme C, Rose KA, Kaminski N (2020). The idiopathic pulmonary fibrosis cell atlas. Am J Physiol Lung Cell Mol Physiol.

[38] Adams TS, Schupp JC, Poli S, Ayaub EA, Neumark N, Ahangari F, Chu SG, Raby BA, DeIuliis G, Januszyk M (2020). Single-cell RNA-seq reveals ectopic and aberrant lung-resident cell populations in idiopathic pulmonary fibrosis. Sci Adv.

[39] Katzen J, Beers MF (2020). Contributions of alveolar epithelial cell quality control to pulmonary fibrosis. J Clin Invest.

[40] Sakamuru S, Attene-Ramos MS, Xia M (2016). Mitochondrial membrane potential assay. Methods Mol Biol.

[41] Bueno M, Calyeca J, Rojas M, Mora AL (2020). Mitochondria dysfunction and metabolic reprogramming as drivers of idiopathic pulmonary fibrosis. Redox Biol.

[42] Cuevas-Mora K, Roque W, Shaghaghi H, Gochuico BR, Rosas IO, Summer R, Romero F (2021). Hermansky–Pudlak syndrome-2 alters mitochondrial homeostasis in the alveolar epithelium of the lung. Respir Res.

[43] Beers MF, Moodley Y (2017). When is an alveolar type 2 cell an alveolar type 2 cell? A Conundrum for lung stem cell biology and regenerative medicine. Am J Respir Cell Mol Biol.

[44] Hans-Willem S, Ya-Wen C, Aabra A. Generation of three-dimensional lung bud organoid and its derived branching colonies. Protoc Exch. 2017.

[45] Zhao M, Liu Y, Liu R, Qi J, Hou Y, Chang J, Ren L (2018). Upregulation of IL-11, an IL-6 family cytokine, promotes tumor progression and correlates with poor prognosis in non-small cell lung cancer. Cell Physiol Biochem.

[46] Grande MT, Sánchez-Laorden B, López-Blau C, De Frutos CA, Boutet A, Arévalo M, Rowe RG, Weiss SJ, López-Novoa JM, Nieto MA (2015). Snail1-induced partial epithelial-to-mesenchymal transition drives renal fibrosis in mice and can be targeted to reverse established disease. Nat Med.

[47] Jonsdottir HR, Arason AJ, Palsson R, Franzdottir SR, Gudbjartsson T, Isaksson HJ, Gudmundsson G, Gudjonsson T, Magnusson MK (2015). Basal cells of the human airways acquire mesenchymal traits in idiopathic pulmonary fibrosis and in culture. Lab Invest.

[48] Wang L, Lyerla T (2010). Histochemical and cellular changes accompanying the appearance of lung fibrosis in an experimental mouse model for Hermansky–Pudlak syndrome. Histochem Cell Biol.

[49] Rock JR, Barkauskas CE, Cronce MJ, Xue Y, Harris JR, Liang J, Noble PW, Hogan BL (2011). Multiple stromal populations contribute to pulmonary fibrosis without evidence for epithelial to mesenchymal transition. Proc Natl Acad Sci U S A.

[50] Kook S, Meng S, Rasmussen ML, Trenary I, Young JD, Gama V, Guttentag SH (2020). Impaired mitochondrial respiration in Hermansky–Pudlak syndrome 1-defective alveolar type 2 cells is associated with enhanced mitochondrial fission. FASEB J.

[51] Nureki SI, Tomer Y, Venosa A, Katzen J, Russo SJ, Jamil S, Barrett M, Nguyen V, Kopp M, Mulugeta S, Beers MF (2018). Expression of mutant Sftpc in murine alveolar epithelia drives spontaneous lung fibrosis. J Clin Invest.

[52] Alysandratos KD, Russo SJ, Petcherski A, Taddeo EP, Acín-Pérez R, Villacorta-Martin C, Jean JC, Mulugeta S, Rodriguez LR, Blum BC (2021). Patient-specific iPSCs carrying an SFTPC mutation reveal the intrinsic alveolar epithelial dysfunction at the inception of interstitial lung disease. Cell Rep.

[53] Chapman HA (2011). Epithelial-mesenchymal interactions in pulmonary fibrosis. Annu Rev Physiol.

