# Supplementary material for: Modeling of lung phenotype of Hermansky–Pudlak syndrome type I using patient-specific iPSCs
Source: Respir Res. 2021 Nov 4;22:284. doi: 10.1186/s12931-021-01877-8 (PMC8570015; doi:10.1186/s12931-021-01877-8)
Supplement: Supplementary file 1 — Additional file 1: Additional material and methods, Fig. S1. Generation of HPS1 patient-specific iPSCs and their gene-corrected counterparts, related to Fig. 1. (A) Teratoma assay of HPS1 patient-specific iPSCs for evaluating pluripotency. Representative images of hematoxylin and eosin staining: ectoderm, neuronal cells; endoderm, gland lumen; mesoderm, cartilage. (Scale bars: 100 μm) (B) Immunofluorescent imaging of representative undifferentiation markers (NANOG, SOX2, OCT3/4, SSEA-4). (Scale bar: 50 μm) (C) TRA1-60 positive cell rate using flow cytometric analysis. (D) Karyotypes of HPS1 patient-specific iPSCs (clone GM14-18) and their gene-corrected counter parts (clone IR9-3). Fig. S2. Isolation of NKX2-1+EpCAM+ cells using CPM as a surface antigen, related to Fig. 2. (A) Flow cytometric analysis of EpCAM, THY1 and CPM in a control cell line after 21-day stepwise differentiation. (B) Flow cytometric analysis of NKX2-1 and CPM in each cell line. (C) Histogram of EpCAM+ and NKX2-1+ cells sorted based on the expression of CPM using MACS. (D) Expression of marker genes in isolated lung progenitor cells of each cell line evaluated by qRT-PCR normalized to that of human adult lung. Data are presented as mean ± SEM (n = 4 from 4 independent experiments). Fig. S3. Validation of phenotypes observed in patient-specific C-LBOs using HPS1KO A549 cells, related to Fig. 2. (A) Sequence data of wild type (WT) and HPS1 knockout (KO) A549 cells. (B) Verification of HPS1 KO at the protein level by immunoblot. Immunoprecipitation was performed to detect the full-length of HPS1 protein. A549 cell lysate transfected with HA-HPS1 was used as a positive control to detect HPS1. (C) Nonsense mediated decay of HPS1 mRNA in HPS1KO A549 cells determined by qRT-PCR, normalized to that of human adult lung. Data are presented as mean ± SEM (n = 3 from 3 independent experiments). Unpaired two-tailed Student’s t test: ***P < 0.001. (D) Expression levels of cytokines (IL11 and TGFB1), transcrip [file 12931_2021_1877_MOESM1_ESM.pdf]

Online Data Supplement

Modeling of lung phenotype of Hermansky-Pudlak syndrome type I using patient-specific iPSCs

Takahiro Suezawa, Shuhei Kanagaki, Yohei Korogi, Kazuhisa Nakao, Toyohiro Hirai, Koji Murakami, Masatoshi Hagiwara, Shimpei Gotoh

SUPPLEMENTAL MATERIAL AND METHODS

Generation and maintenance of HPS1 patient-specific iPSCs

GM14609 HPS1 patient-derived fibroblasts were obtained from the Coriell institute for medical research and reprogramed to iPSCs as described previously [1]. Reprograming episomal vectors carrying OCT3/4, SOX2, KLF4, L-MYC, LIN28 and p53-shRNA were electroporated into fibroblasts. The cells were harvested several days after transfection and reseeded on mitomycin c (Kyowa Kirin)-treated feeder STO fibroblasts (DS Parma Biomedical). From the next day, primate ES medium (ReproCELL) supplemented with 4 ng/mL FGF-2 (DS Pharma Biomedical) was used for culture. About one month post-transfection, the iPSC colonies were picked up. The cloned HPS1 patient-specific iPSCs were passaged several times in feeder-dependent culture and then switched to feeder-free culture using Essential 8 medium (Thermo Fisher Scientific), according to the manufacturer's protocol.

Teratoma formation assay

Under approval by Animal Research Committee of Kyoto University, 5×10^5 HPS1 patient-specific iPSCs suspended in DMEM/F12 (Thermo Fisher Scientific) supplemented with 10 μM Y-27632 (LC laboratories) were injected into the testis of 8-week-old SCID mice (CLEA-Japan), and sacrificed three months later. Tumors were harvested and fixed using Mildform 10N (FUJIFILM Wako), embedded in paraffin, and sectioned for hematoxylin and eosin (H&E) staining. Bright field images were obtained using BZ-X710 microscope (Keyence).

Gene correction of HPS1 patient-specific iPSCs

A guide RNA (gRNA) was designed to cause a double-strand break in the 16 bp duplication in HPS1 patient-specific DNA sequence with the most frequent genotype, occurring at over 80%, in the batch mode

analysis of the inDelphi software program (gRNA sequence: GCAGGGGAGGCCCCCAGCAG, sense strand) [2]. This gRNA sequence was consistent with that of another group [3]. gRNA was synthesized (Macrogen) and cloned into pHL-H1-ccdB-mEF1a-RIH. pHL-H1-ccdB-mEF1a-RiH was a gift from Dr. Akitsu Hotta (Addgene plasmid # 60601; http://n2t.net/addgene:60601; RRID:Addgene_60601) [4]. To perform single-cell cloning, the medium of HPS1 patient-specific iPSCs was switched from Essential 8 medium to StemFit AK02N (Ajinomoto) and cultured for 3 weeks according to the manufacturer's protocol. Prior to electroporation, the cells were harvested with TrypLE select (Thermo Fisher Scientific), 1:2 diluted with 0.5 mM EDTA/PBS. Approximately 1×10^6 HPS1 patient-specific iPSCs were electroporated with 5 μg each of spCas9 expression vector, pHL-EF1a-SphcCas9-iP-A, and the gRNA expression vector using NEPA21 (Nepa Gene) as described previously [1, 5]. pHL-EF1a-SphcCas9-iP-A was a gift from Dr. Akitsu Hotta (Addgene plasmid # 60599; http://n2t.net/addgene:60599; RRID:Addgene_60599) [4]. Electroporated cells were suspended in StemFit AK02N supplemented with 10 μM Y-27632 and seeded on a dish coated with iMatrix-511 (Matrixome). One day after seeding, the medium was replaced with StemFit AK02N and the cells were cultured for another 2 days. The cells were harvested with 1:2 diluted TrypLE and suspended in PBS containing 1% BSA (Thermo Fisher Scientific), 10 μM Y-27632, and SYTOX Blue Dead Cell Stain (Thermo Fisher Scientific). The cells that transiently expressed mRFP1 were sorted by FACSAriaIII (BD Biosciences). Cells sorted by fluorescence-activated cell sorting (FACS) were cloned by limiting dilution in 96-well plates.

PCR analysis of the 16 bp duplication in HPS1 patient-specific genomic DNA

Genomic DNA was extracted from the cloned cells using PureLink Genomic DNA Mini Kit (Thermo Fisher Scientific) according to the manufacturer's protocol. Touch down PCR was performed using KOD -Plus- NEO (TOYOBO) with a forward primer, 5'-GGTCCCTTCTGCTGTAATGC-3', and a reverse

primer, 5’-GCTGCGTGAAGGAAGTACG-3’, for the exon 15 of *HPS1* gene (amplicon size: healthy donor, 240 bp; HPS1 patient, 256 bp) [6, 7] with the thermal cycling condition as follows: 94°C for 2 min; 98 °C for 10 sec, and 74 °C for 20 sec in 5 cycles; 98 °C for 10 sec and 72 °C for 20 sec in 5 cycles; 98 °C for 10 sec and 70 °C for 20 sec in 5 cycles; 98 °C for 10 sec and 68 °C for 20 sec in 30 cycles; 68 °C for 5 min. Each PCR product was loaded on 5% polyacrylamide Mini Protien TBE precast gel (Bio-Rad) and electrophoresed at 100 V for 1 h in TBE buffer (Bio-Rad). The gels were stained with Midori Green Advance (NIPPON genetics) and analyzed using ChemiDoc Touch MP (Bio-Rad). Out of 92 clones, 3 had a single amplicon of approximately the same size as the healthy-donor-derived control iPSCs and were selected for downstream analysis. These amplicons were treated with ExoSAP-IT Express (Thermo Fisher Scientific) and sequenced to confirm the gene correction.

Karyotyping

Karyotypes of HPS1 patient-specific-iPSCs (clone: GM14-18) and gene-corrected iPSCs (clone: IR9-3) were determined by G-banding by Nihon Gene Research Laboratories Inc, Japan.

Immunofluorescence

All specimens were fixed in 4% paraformaldehyde/PBS (Nacalai Tesque): 15 min for 2D cultured iPSCs and 30 min for 3D cultured organoids. For organoids, fixed specimens were immersed in 30% sucrose (Nacalai Tesque) overnight at 4 °C, embedded in optimal cutting temperature (OCT) compound (Sakura Finetek) and frozen at −80 °C, and sectioned into 10-μm. Permeabilization was performed using PBS containing 0.2% Triton X-100 (Nacalai Tesque) or ice-cold methanol (Nacalai Tesque) for 15 min. Permeabilized specimens were immersed in the blocking buffer of PBS containing 5% normal donkey serum (EMD-Millipore) and 1% BSA (Sigma-Aldrich) for 30 min. They were immunostained with the

primary antibodies overnight at 4 °C and secondary antibodies for 1 h. The staining conditions for each antibody are listed in Table S1 and S2. Blocking buffer supplemented with 0.1% Hoechst 33342 (Dojindo) was used for dilution of secondary antibodies. For organoids, each section was mounted in ProLong Gold Antifade Mountant (Thermo Fisher Scientific) and imaged using BZ-X710 microscope or TCS SP8 confocal microscope (Leica Microsystems).

Flow cytometry

PBS containing 1% BSA and 10 μM Y-27632 was used for cell suspension, washing and antibody dilution. FACSAriaIII was used for all FACS analysis in the present study. Cells were dissociated using Accutase (Innovative Cell Technologies) for undifferentiated iPSCs in 2D culture or 0.1% Trypsin/EDTA (Thermo Fisher Scientific) for organoids, as described previously [8]. For intracellular staining, they were fixed with 4% paraformaldehyde/PBS for 15 min and permeabilized with ice-cold methanol at −30 °C for at least 30 min. The cells were immunostained with primary and secondary antibodies at 4 °C for 20 min. The staining conditions for each antibody are listed in Table S1 and S2. For live cell staining, dead cells were removed using Propidium iodide (Dojindo) or SYTOX Blue Dead Cell Stain.

Western blotting

HPS1-overexpressing A549 cell extracts were prepared as positive controls for the detection of full-length HPS1. A549 cells were obtained from ATCC and cultured as described previously [9]. N-terminally HA-tagged HPS1 cDNA cloned into pCAG-Neo (Fujifilm Wako) was transfected into A549 cells using the Lipofectamine LTX Reagent (Thermo Fisher Scientific), according to the manufacture's protocol. Transfected A549 cells and undifferentiated iPSCs were lysed with the Pierce IP Lysis Buffer (Thermo Fisher Scientific) supplemented with cOmplete™ Mini Protease Inhibitor Cocktail (Merck). Each lysate

was centrifuged at 15,000 × *g* for 15 min and supernatant was collected. Protein concentration was determined using Pierce BCA Protein Assay Kit (Thermo Fisher Scientific), and protein concentration and volume were matched among samples (5 mg/mL, 1 mL for iPSCs). For detection of HPS4 and β-Actin, about 5% of each lysate was separated, added 1/4th volume of 4X Laemmli sample buffer (supplemented with 2-mercaptoethanol; Bio-Rad) relative to lysate volume, and boiled for 5 min. Two microgram of mouse anti-human HPS1 antibody (Santa Cruz Biotechnology #sc-101435) was added into each of the remaining cell lysates and incubated overnight with rotation at 4 °C. Protein G Mag Sepharose Xtra (Cytiva) magnetic beads were washed, resuspended in 50 μL/sample of Pierce IP Lysis Buffer, added to each cell lysate and incubated with rotation for 1 h. After three cycles of magnetic capture with the sepharose beads and resuspension in PBS containing 0.1% BSA and 2 mM EDTA, the supernatant was completely removed from each sample using a magnet. Laemmli sample buffer (4X) supplemented with 2-mercaptoethanol was diluted to 2X with Pierce IP Lysis Buffer. Then, 30 μL of buffer was added to each sample with sepharose beads and boiled for 5 min. Supernatants were collected using a magnet and were used to detect HPS1. Each sample was loaded on polyacrylamide (7.5% for HPS1, 4–20% for HPS4 and β-Actin) Mini Protien TGX precast gels (Bio-Rad), electrophoresed in Tris Glycine SDS buffer (Bio-Rad) at 130 V for 90 min, and transferred to methanol-activated Immobilon-P polyvinylidene difluoride (PVDF) membrane (Merck, 0.2 μm) in Tris Glycine buffer (Bio-Rad) supplemented with 20% methanol at 100 V for 90 min at 4 °C. The membranes were immersed in TBS-T (Takara Bio) containing 3% BSA for HPS1 and β-Actin, or 1:2 diluted Blocking One (Nacalai Tesque) with TBS-T for HPS4, and incubated for 1 h. Next, the membranes were immunostained with primary antibodies overnight at 4 °C. After three washings with TBS-T, they were immunostained with HRP-conjugated secondary antibodies for 1 h. The staining conditions for each antibody are listed in Table S1 and S2. Can Get Signal Solution 2 (Toyobo) was used for dilution of secondary antibodies. Pierce ELC plus Western Blotting Substrate (Thermo Fisher

Scientific) was used for detection of chemiluminescence, according to the manufacturer's protocol. Images were obtained using ChemiDoc Touch MP.

qRT-PCR

Total RNA was extracted with PureLink RNA Mini Kit (Thermo Fisher Scientific), and cDNA was synthesized with ReverTra Ace qPCR RT Master Mix with gDNA Remover (Toyobo), according to the manufacturer's protocol. The primers used in this study are listed in Tables S3 and S4. TaqPath qPCR Master Mix, CG (Thermo Fisher Scientific), or Power SYBR Green Master Mix (Thermo Fisher Scientific) was used for qPCR in the StepOnePlus Real-Time PCR System (Thermo Fisher Scientific) or ABI7300 Real-Time PCR System (Thermo Fisher Scientific). Each gene expression was normalized using the eukaryotic 18S rRNA, and was presented as a relative value to the human adult lung 5 donor pool (BioChain #R1234152-P, lot A811037).

Differentiation of iPSCs into NKX2-1$^+$ lung epithelial progenitor cells

iPSCs cultured in Essential 8 medium or mTeSR plus (STEMCELL Technologies) for more than 2 weeks were differentiated into NKX2-1$^+$ lung epithelial progenitor cells, as previously described [8]. Briefly, 1.2–1.5 × 10^6 healthy-donor derived iPSCs (B2-3) or 0.9 × 10^6 HPS1 patient specific- and gene corrected-iPSCs were seeded on Geltrex (Thermo Fisher Scientific) -coated 6-well plates (Greiner Bio-One) in Roswell Park Memorial Institute (RPMI) medium (Nacalai Tesque) supplemented with 100 ng/mL Activin A (Peprotech), 1 μM CHIR99021 (Axon Medchem), 10 μM Y-27632, 2% B-27 supplement (Thermo Fisher Scientific), and 50 U/mL penicillin-streptomycin (Thermo Fisher Scientific) to differentiate into definitive endoderm (DE). The medium was replaced every other day. Sodium butyrate (Fujifilm Wako) was added to the medium at a final concentration of 0.25 mM on the next day and switched to 0.125 mM

for another 4 days to obtain DE cells. Basal medium, comprising DMEM/F12, 2% B-27 supplement, 50 U/mL penicillin-streptomycin, 0.05 mg/mL L-ascorbic acid (Fujifilm Wako), 1% GlutaMax (Thermo Fisher Scientific), and 0.4 mM monothioglycerol (Fujifilm Wako), was used in the following steps. The DE cells were differentiated into anterior foregut endoderm (AFE) cells by culturing for 4 days in the basal medium supplemented with 100 ng/mL Noggin (Proteintech) and 10 μM SB431542 (Fujifilm Wako). The AFE cells were differentiated into ventralized anterior foregut endoderm (VAFE) cells by culturing for 4 days in the basal medium supplemented with 3 μM CHIR99021, 20 ng/mL BMP4 (Proteintech), and 0.05 μM all-trans retinoic acid (Sigma-Aldrich) for healthy-donor derived iPSCs or 0.5 μM for HPS1 patient-specific and gene-corrected iPSCs. The VAFE cells were cultured for 7 days in the basal medium supplemented with 3 μM CHIR99021, 10 ng/mL FGF10 (PeproTech), 10 ng/mL keratinocyte growth factor (KGF) (PeproTech) and 20 μM DAPT (Fujifilm Wako) for efficient distalization. After the 21-day differentiation step, NKX2-1$^+$ lung epithelial progenitor cells were enriched using CPM as the cell surface antigen through magnetic-activated cell sorting (MACS) or FACS, as described previously [10]. The staining conditions for each antibody are listed in Table S1 and S2.

Generation of CPM-isolated lung bud organoids

CPM-isolated lung bud organoids (C-LBOs) were cultured in the LBO medium: Iscove's modified Dulbecco's medium (IMDM; Fujifilm Wako) and Ham's F-12 (Fujifilm Wako) were mixed in 3:1 ratio and supplemented with 0.5% N-2 supplement (Thermo Fisher Scientific), 1% B-27 supplement, 50 U/mL penicillin-streptomycin, 0.05 mg/mL L-ascorbic acid, 1% GlutaMax, 0.4 mM monothioglycerol, 3 μM CHIR99021, 10 ng/mL BMP4, 10 ng/mL FGF10, and 50 nM all-trans retinoic acid. Isolated 5×10^3 CPM$^+$ lung epithelial progenitor cells were suspended in the LBO medium supplemented with 10 μM Y-27632, seeded into Prime Surface 96U plate (Sumitomo Bakelite), and centrifuged at $100 \times g$ for 2 min on Day

0. They were incubated for 7 days to form a spheroid in each well with 50% medium replaced every other day. On Day 7, a spheroid collected from each well was mixed with 2-3 spheroids in a tube, and they were allowed to settle spontaneously and the supernatant was removed. Next, 50 μL of ice-cold growth factor reduced Matrigel (Corning) was placed in a 24-well cell culture insert (Corning) and allowed to solidify at 37 °C for 5 min forming a bottom layer of organoid culture. About 2-3 spheroids were resuspended in 30 μL of Matrigel, placed onto the bottom layer, and incubated at 37 °C for 5 min, forming an organoid layer. Additional 50 μL of Matrigel was added onto the organoid layer and solidified at 37 °C for 5 min, forming a top layer. Then, 500 μL of LBO medium supplemented with 10 μM Y-27632 was added to the lower chamber. The medium in the lower chamber was replaced with LBO medium every 2-3 days until Day 35.

Generation of HPS1KO A549 cells

A549 cells were obtained from ATCC and cultured as described previously. gRNA was designed to cause a double-strand break in exon 5 of *HPS1* gene (gRNA sequence: GGACGGTCATCTTATCCGAA, sense strand). gRNA was synthesized (Macrogen) and cloned into pHL-H1-ccdB-mEF1a-RIH. The gRNA expression vector and the spCas9 expression vector, pHL-EF1a-SphcCas9-iP-A, were transfected into A549 cells using the FuGENE HD Transfection Reagent (Promega), according to the manufacture's protocol. One day after transfection, the transfected cells were cultured in the culture medium containing 200 μg/mL hygromycin B (Nacakai Tesque) for another 9 days, and colonies were picked up. Genomic DNA was extracted from the cloned cells using PureLink Genomic DNA Mini Kit and amplified using KOD -Plus- NEO with a forward primer, 5'-TTTTTGCTCCCCTCCCTACC-3', and a reverse primer, 5'-TGGGAGGATGGAAAGAGCAC-3'. The amplicon was cloned into pTA2 vector (TOYOBO) according to the manufacture's protocol and sequenced to confirm frameshifts of *HPS1* gene with a sequencing

primer 5’-CCCAGGCAAGTGAGTTCCAT-3’.

Formation and maintenance of alveolar organoids.

Human fetal lung fibroblasts (HFLFs) (17.5 weeks of gestation; DV Biologics #PP002-F-1349, lot 121109VA) were cultured in DMEM (Nacalai Tesque) containing 10% fetal bovine serum (Sigma-Aldrich #F7524) and 50 U/mL penicillin-streptomycin, and used at passage 10 for the formation of alveolar organoids (AOs) [8]. They were cultured as described previously in the DCIK medium: Ham's F12 containing 1% B-27 supplement, 0.25% BSA, 15 mM HEPES (Thermo Fisher Scientific), 0.8 mM $CaCl_2$ (Fujifilm Wako), 0.1% ITS premix (Corning), 50 U/mL penicillin-streptomycin, 50 nM dexamethasone (Sigma-Aldrich), 100 μM 8-Br-cAMP (Biolog Life Science Institute), 100 μM 3-isobutyl-1-methylxanthine (Fujifilm Wako), and 10 ng/mL KGF [8]. Isolated 1×10^4 CPM^+ lung epithelial progenitor cells were mixed with 5×10^5 HFLFs in 200 μL of 1:2 diluted Matrigel with DCIK medium supplemented with 10 μM Y-27632, and placed in a 12-well cell culture insert (Corning). One milliliter of DCIK medium containing 10 μM Y-27632 was added to the lower chamber. The medium in the lower chamber was replaced with DCIK medium every 2-3 days. $NaPi2B^+$ or GFP-SPC^+ cells were isolated every 14 days and continuously cultured in AOs to maintain and allow the maturation of AT2 cells, as described previously [1, 8].

Measurement of organoid sizes

Live cell images of C-LBOs and AOs were obtained using the BZ-X710 microscope. Images were combined, and the diameter of each organoid was measured using BZ-X Analyzer (Keyence). All 3 C-LBOs in a well were measured in 4 independent experiments, whereas 20 randomly selected AOs in a well were measured in 6 independent experiments.

RNA-seq

Total RNAs of FACS-sorted NaPi2B^{high} or SPC-GFP^{+} cells from AOs were extracted and DNA was removed using the RNeasy micro kit (Qiagen), according to the manufacturer's protocol. The RNA integrity number calculated by 2100 BioAnalyer (Agilent Technologies) for each sample was confirmed to be > 8.0. Sequence libraries for NaPi2B^{high} cells were synthesized using the TruSeq Stranded mRNA (illumina). For SPC-GFP^{+} cells, 10 ng of total RNA was reverse-transcribed, amplified with 7 cycles using SMART-Seq v4 Ultra Low Input RNA Kit for Sequencing (Clontech Laboratories), and converted to sequence libraries using Nextera XT DNA Library Preparation Kit (Illumina). The library was sequenced using 100 bp paired-end method by NovaSeq 6000 (Illumina).

Bioinformatic analysis of RNA-seq data

Publicly available fastq files were downloaded from Sequence Read Archive: GSE163575, lung epithelial cell progenitors; GSE121999, LBOs; GSE131768, adult AT2 cells; GSE94555, AT2 cells from idiopathic pulmonary fibrosis (IPF). Sequenced reads were trimmed using fastp [11] and aligned to the GRCh38 using STAR 2.7.3a [12]. RSEM was used to calculate transcripts per million (TPM) values [13]. Low expression genes, with total TPM values of the corresponding gene expression in the data set under 6, were excluded for listing differentially expressed genes (DEGs) and conducting principal component analysis (PCA). R package DESeq2 [14] was used to identify DEGs, and the volcano plot was visualized using R package "ggplot2". Gene ontology (GO) enrichment analysis was performed using the PANTHER online software [15]. PCA of $\log_2$ (TPM + 0.01) was performed using the R function "prcomp", and the results were visualized using the R package "ggbiplot". A hierarchical clustering for $\log_2$ (TPM + 0.01) of lung epithelial cell markers was performed using "heatmap.2" in the R package "gplots".

Proteomic analysis

AO cells were carefully dissociated with 0.1% Trypsin-EDTA, as described previously [8]. The cells were immunostained with mouse anti-human EpCAM antibody (Santa Cruz Biotechnology #sc-66020) and goat anti-mouse IgG microbeads (Miltenyi Biotec #130-048-401) at 4°C for 20 min. MACS was performed using LS columns (Miltenyi Biotec) to obtain $EpCAM^+$ cells. The retrieved cells were washed twice with PBS and flash frozen in liquid nitrogen. First, 100 µL of lysis reagent (10 mM Tris-HCl buffer pH 8.0 (Cytiva), 7 M urea (Cytiva), 2 M thiourea (Fujifilm Wako), 5 mM magnesium acetate (Fujifilm Wako), 4% (w/v) CHAPS (Dojindo) with Complete Protease Inhibitor Cocktail (Roche)) was added to each sample. Each sample was sonicated and centrifuged at 20,000 × *g* for 30 min to obtain supernatants, which were mixed with 100 µL of reducing reagent (10 mM DTT (Cytiva) and 100 mM ammonium bicarbonate (Fujifilm Wako)) and incubated at 57 °C for 30 min. Next, 100 µL of alkylation reagent (50 mM iodoacetamide (Sigma-Aldrich) and 100 mM ammonium bicarbonate) was added to the samples and incubated for 30 min. Further, first, 100 µL of Sequencing Grade Modified Trypsin (Promega) and then 100 µL of 50 mM ammonium bicarbonate were added to each sample, and the sample were incubated at 30 °C for 16 h and dried in a centrifugal concentrator (TOMY #CC-105). Subsequently, each dried sample was dissolved in 30 µL of 0.1% formic acid (Fujifilm Wako) and centrifuged at 20,000 × *g* for 10 min, and the supernatants were used for nanoLC-MS/MS in the UltiMate 3000 liquid chromatograph (Thermo Fisher Scientific) and analyzed using the Q-Exactive Plus mass spectrometer (Thermo Fisher Scientific). Mascot software program (Matrix Science) was used to search the peptide sequence database, and the results were analyzed using Scaffold software program (Proteome Software). Data independent acquisition (DIA) analysis was performed using a BLIB file exported from the Scaffold software program analyzing nanoLC-MS/MS data. Protein expression was determined twice as technical replicates in each

sample. Then, the acquired MS/MS data were annotated as human proteins by searching in Uni-Prot [16] and quantified using the Scaffold DIA software program (Proteome Software). Next, 3098 proteins with false discovery rate < 0.01 were used for downstream analysis. GO enrichment analysis was performed using the PANTHER online software for upregulated proteins with $\log_2$ (fold change) > 0.5, or downregulated proteins with $\log_2$ (fold change) < −0.5. GSEAPreranked test was run using the genes ordered by $\log_2$ (fold change) [17].

Evaluation of mitochondrial membrane potential

AO cells were carefully dissociated with 0.1% Trypsin-EDTA, as described previously. The dissociated cells were stained with HBSS (+) (Nacalai tesque) containing 250 nM tetramethylrhodamine, ethyl ester (TMRE) (Thermo Fisher Scientific) and APC-conjugated mouse anti EpCAM-APC antibody for 30 min at 37℃. The stained cells were washed twice with HBSS (+) containing 1%BSA and 10 μM Y-27632, and analyzed using FACSAriaIII.

Evaluation of intracellular reactive oxygen species (ROS) levels

Two-dimensional cultured A549 cells were stained with highly sensitive DCFH-DA Dye (Dojindo) and 200 nM MitoTracker Deep Red FM (Thermo Fisher Scientific), as per the manufacture's protocol. The stained cells were washed twice with HBSS (+) and analyzed using BZ-X710 microscope or FACSAriaIII.

Transmission electron microscope

Lamellar bodies were examined using transmission electron microscope (Hitachi; H-7650) as described previously [9]. Briefly, AOs were incubated in a fixative solution, en bloc stained, and embedded in Epon 812 (Nacalai Tesque). Uranyl acetate and lead citrate were used to stain the ultrathin sections.

SUPPLEMENTAL REFERENCES

1. Korogi Y, Gotoh S, Ikeo S, Yamamoto Y, Sone N, Tamai K, Konishi S, Nagasaki T, Matsumoto H, Ito I, et al: **In Vitro Disease Modeling of Hermansky-Pudlak Syndrome Type 2 Using Human Induced Pluripotent Stem Cell-Derived Alveolar Organoids.** *Stem Cell Reports* 2019, **12:**431-440.
2. Shen MW, Arbab M, Hsu JY, Worstell D, Culbertson SJ, Krabbe O, Cassa CA, Liu DR, Gifford DK, Sherwood RI: **Predictable and precise template-free CRISPR editing of pathogenic variants.** *Nature* 2018, **563:**646-651.
3. Iyer S, Suresh S, Guo D, Daman K, Chen JCJ, Liu P, Zieger M, Luk K, Roscoe BP, Mueller C, et al: **Precise therapeutic gene correction by a simple nuclease-induced double-stranded break.** *Nature* 2019, **568:**561-565.
4. Li HL, Fujimoto N, Sasakawa N, Shirai S, Ohkame T, Sakuma T, Tanaka M, Amano N, Watanabe A, Sakurai H, et al: **Precise correction of the dystrophin gene in duchenne muscular dystrophy patient induced pluripotent stem cells by TALEN and CRISPR-Cas9.** *Stem Cell Reports* 2015, **4:**143-154.
5. Li HL, Gee P, Ishida K, Hotta A: **Efficient genomic correction methods in human iPS cells using CRISPR-Cas9 system.** *Methods* 2016, **101:**27-35.
6. Korbie DJ, Mattick JS: **Touchdown PCR for increased specificity and sensitivity in PCR amplification.** *Nat Protoc* 2008, **3:**1452-1456.
7. Kirshenbaum AS, Cruse G, Desai A, Bandara G, Leerkes M, Lee CC, Fischer ER, O'Brien KJ, Gochuico BR, Stone K, et al: **Immunophenotypic and Ultrastructural Analysis of Mast Cells in Hermansky-Pudlak Syndrome Type-1: A Possible Connection to**

Pulmonary Fibrosis. *PLoS One* 2016, **11:**e0159177.

8. Yamamoto Y, Gotoh S, Korogi Y, Seki M, Konishi S, Ikeo S, Sone N, Nagasaki T, Matsumoto H, Muro S, et al: **Long-term expansion of alveolar stem cells derived from human iPS cells in organoids.** *Nat Methods* 2017, **14:**1097-1106.

9. Kanagaki S, Suezawa T, Moriguchi K, Nakao K, Toyomoto M, Yamamoto Y, Murakami K, Hagiwara M, Gotoh S: **Hydroxypropyl Cyclodextrin Improves Amiodarone-induced Aberrant Lipid Homeostasis of Alveolar Cells.** *Am J Respir Cell Mol Biol* 2021, **64:**504-514.

10. Konishi S, Gotoh S, Tateishi K, Yamamoto Y, Korogi Y, Nagasaki T, Matsumoto H, Muro S, Hirai T, Ito I, et al: **Directed Induction of Functional Multi-ciliated Cells in Proximal Airway Epithelial Spheroids from Human Pluripotent Stem Cells.** *Stem Cell Reports* 2016, **6:**18-25.

11. Chen S, Zhou Y, Chen Y, Gu J: **fastp: an ultra-fast all-in-one FASTQ preprocessor.** *Bioinformatics* 2018, **34:**i884-i890.

12. Dobin A, Davis CA, Schlesinger F, Drenkow J, Zaleski C, Jha S, Batut P, Chaisson M, Gingeras TR: **STAR: ultrafast universal RNA-seq aligner.** *Bioinformatics* 2013, **29:**15-21.

13. Li B, Dewey CN: **RSEM: accurate transcript quantification from RNA-Seq data with or without a reference genome.** *BMC Bioinformatics* 2011, **12:**323.

14. Love MI, Huber W, Anders S: **Moderated estimation of fold change and dispersion for RNA-seq data with DESeq2.** *Genome Biol* 2014, **15:**550.

15. Mi H, Ebert D, Muruganujan A, Mills C, Albou LP, Mushayamaha T, Thomas PD: **PANTHER version 16: a revised family classification, tree-based classification tool,**

enhancer regions and extensive API. *Nucleic Acids Res* 2021, **49:**D394-d403.

16. **UniProt: the universal protein knowledgebase in 2021.** *Nucleic Acids Res* 2021, **49:**D480-d489.

17. Subramanian A, Tamayo P, Mootha VK, Mukherjee S, Ebert BL, Gillette MA, Paulovich A, Pomeroy SL, Golub TR, Lander ES, Mesirov JP: **Gene set enrichment analysis: a knowledge-based approach for interpreting genome-wide expression profiles.** *Proc Natl Acad Sci U S A* 2005, **102:**15545-15550.

SUPPLEMENTAL FIGURES AND LEGENDS

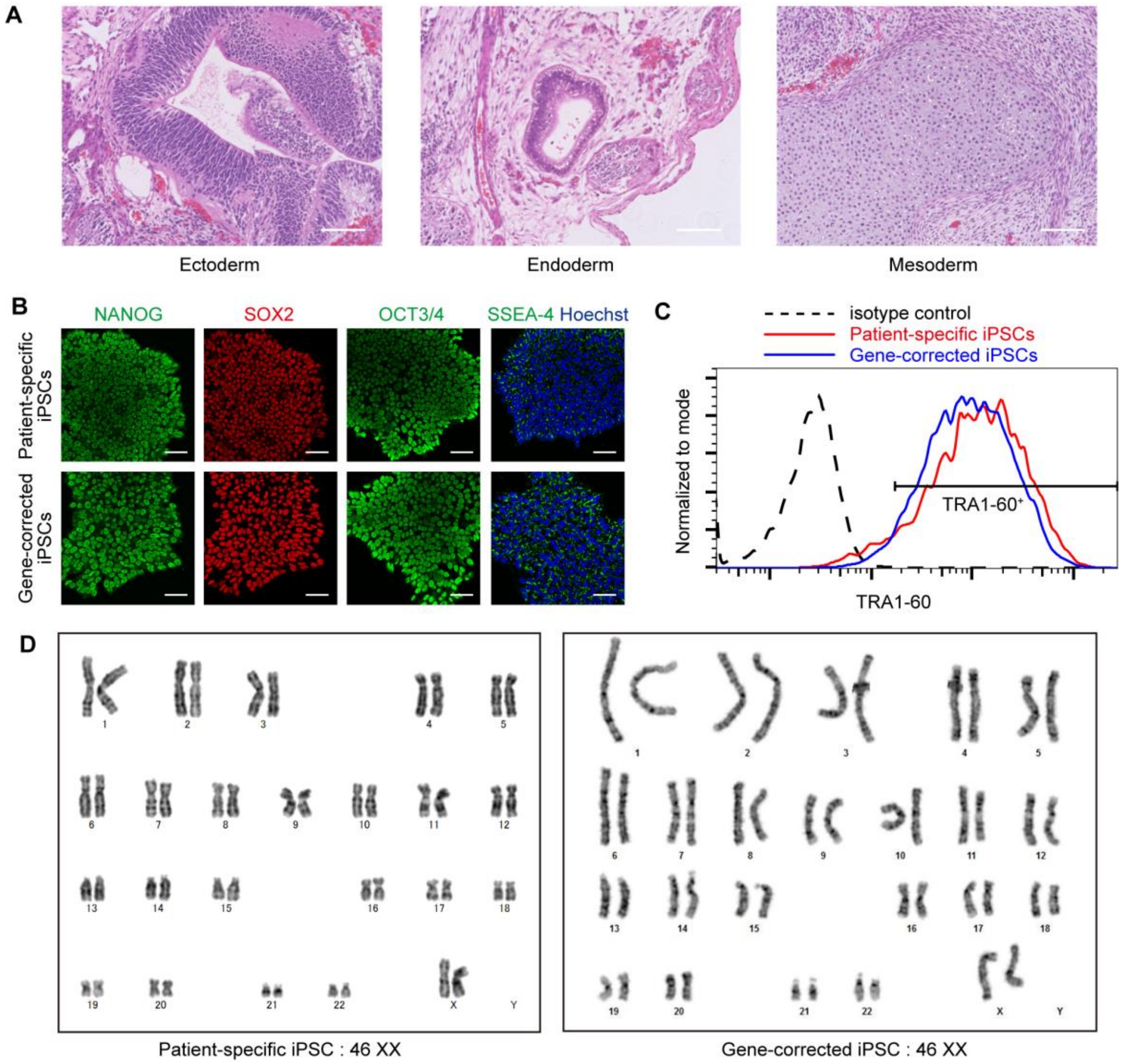

Fig. S1 Generation of HPS1 patient-specific iPSCs and their gene-corrected counterparts, related to Fig. 1

(A) Teratoma assay of HPS1 patient-specific iPSCs for evaluating pluripotency. Representative images of hematoxylin and eosin staining: ectoderm, neuronal cells; endoderm, gland lumen; mesoderm, cartilage. (Scale bars: 100 µm) (B) Immunofluorescent imaging of representative undifferentiation markers (NANOG, SOX2, OCT3/4, SSEA-4). (Scale bars: 50 µm) (C) TRA1-60 positive cell rate using flow cytometric analysis. (D) Karyotypes of HPS1 patient-specific iPSCs (clone GM14-18) and their gene-corrected counter parts (clone IR9-3).

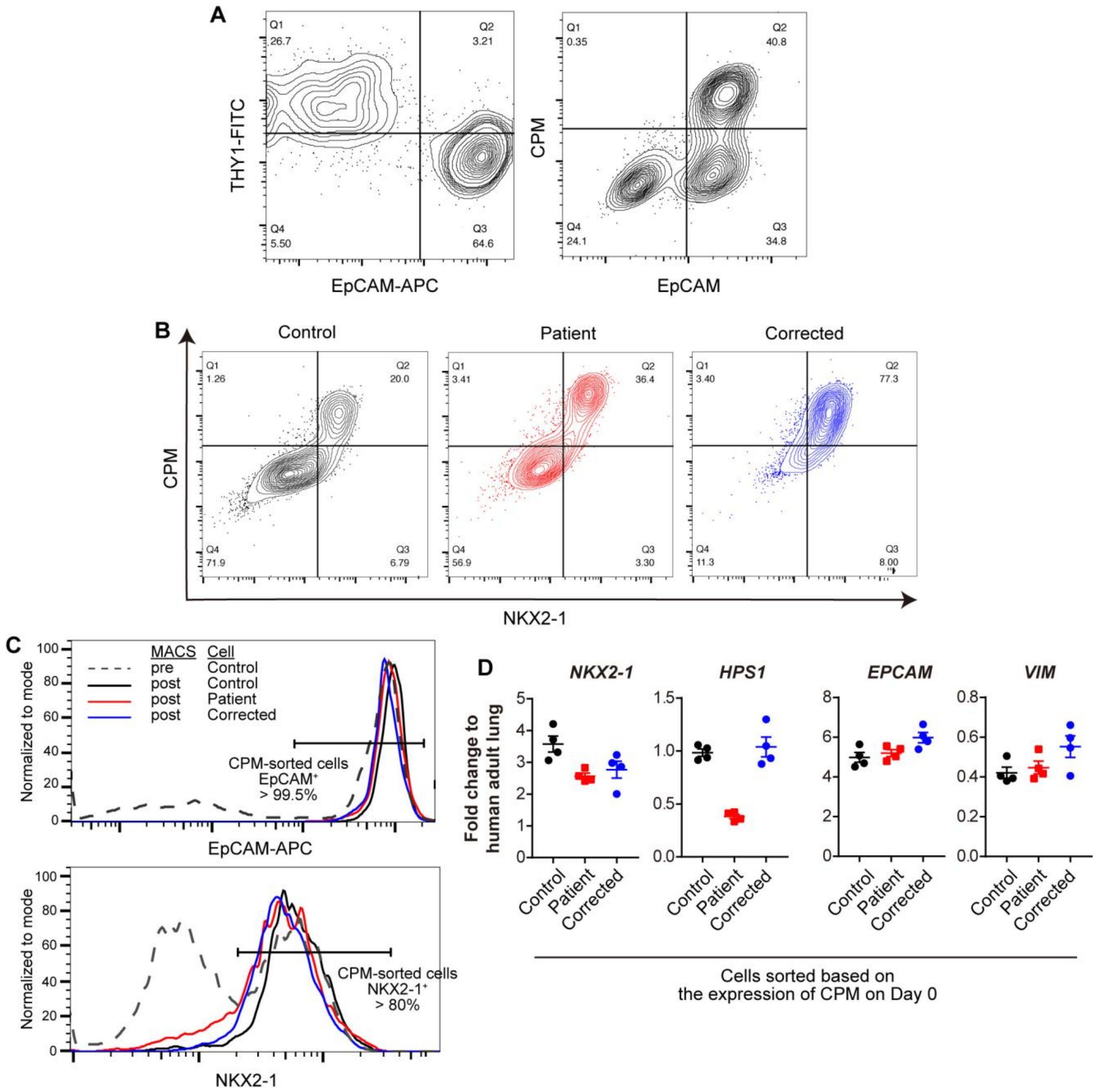

Fig. S2 Isolation of NKX2-1$^+$EpCAM$^+$ cells using CPM as a surface antigen, related to Fig. 2

(A) Flow cytometric analysis of EpCAM, THY1 and CPM in a control cell line after 21-day stepwise differentiation. (B) Flow cytometric analysis of NKX2-1 and CPM in each cell line. (C) Histogram of EpCAM$^+$ and NKX2-1$^+$ cells sorted based on the expression of CPM using MACS. (D) Expression of marker genes in isolated lung progenitor cells of each cell line evaluated by qRT-PCR normalized to that of human adult lung. Data are presented as mean ± SEM (n = 4 from 4 independent experiments).

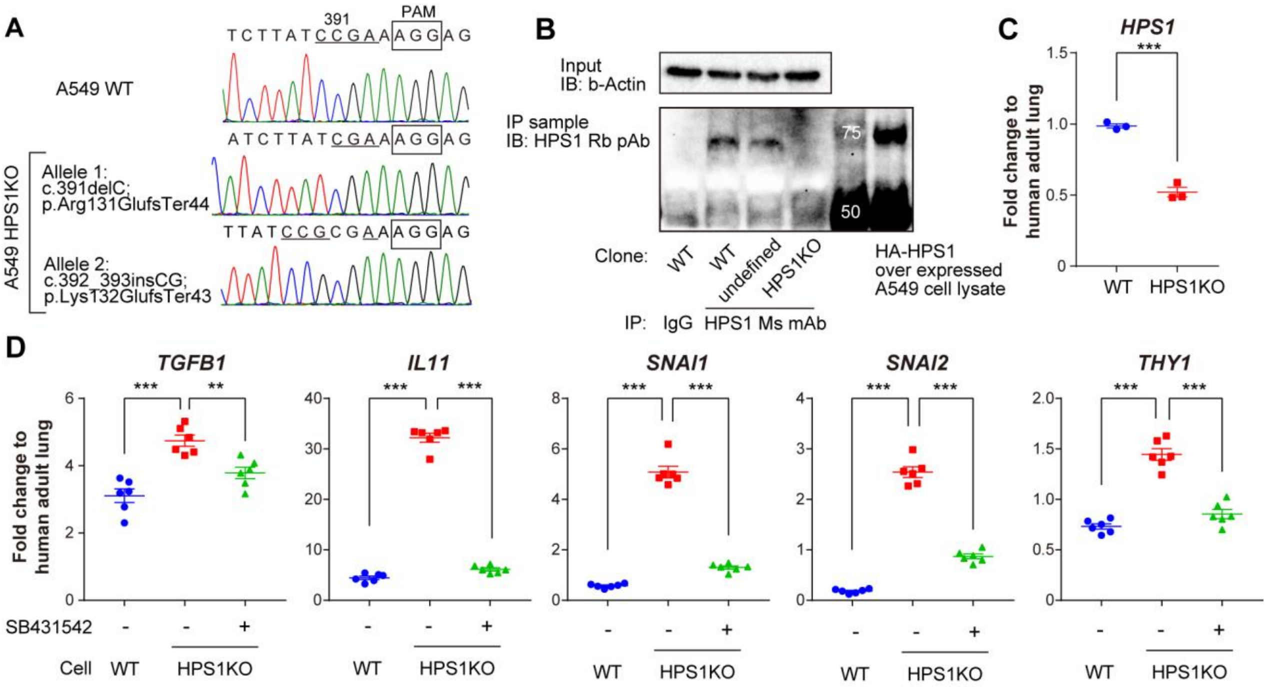

Fig. S3 Validation of phenotypes observed in patient-specific C-LBOs using HPS1KO A549 cells, related to Fig. 2

(A) Sequence data of wild type (WT) and HPS1 knockout (KO) A549 cells. (B) Verification of HPS1 KO at the protein level by immunoblot. Immunoprecipitation was performed to detect the full-length of HPS1 protein. A549 cell lysate transfected with HA-HPS1 was used as a positive control to detect HPS1. (C) Nonsense mediated decay of HPS1 mRNA in HPS1KO A549 cells determined by qRT-PCR, normalized to that of human adult lung. Data are presented as mean ± SEM (n = 3 from 3 independent experiments). Unpaired two-tailed Student's t test: ****P* < 0.001. (D) Expression levels of cytokines (*IL11* and *TGFB1*), transcription factors (*SNAI1* and *SNAI2*), and a mesenchymal cell marker (*THY1*) in HPS1 KO A549 cells, treated with 10 μM SB431542 for 3 days determined by qRT-PCR, normalized to that of human adult lung. Data are presented as mean ± SEM (n = 6 from 3 independent experiments). One-way ANOVA with Tukey's multiple comparisons test: ***P* < 0.01, ****P* < 0.001.

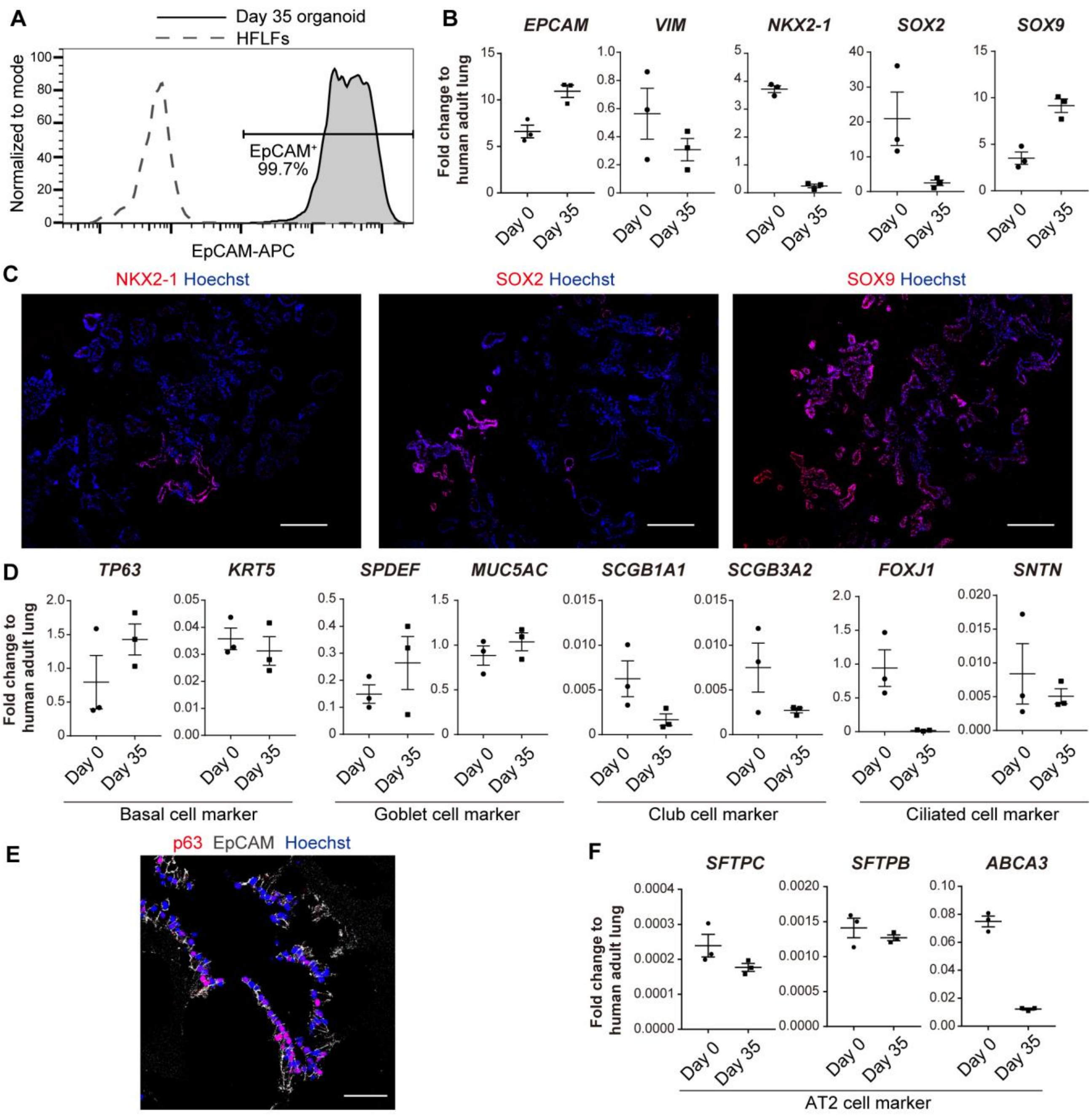

Fig. S4 Analysis of cell lineages in CPM-isolated lung bud organoids derived from healthy-donor derived control iPSCs on Day 35, related to Fig. 2

(A) Ratio of $EpCAM^{+}$ cells on Day 35 by flow cytometric analysis. HFLFs were used as a negative control.

(B) Gene expression of the pan-epithelial (*EPCAM*), pan-mesenchymal (*VIM*), and transcription factors related to lung development (*NKX2-1*, *SOX2*, and *SOX9*) determined by qRT-PCR, normalized to that of

human adult lung (n = 3 from 3 independent experiments). (C) Immunofluorescent images on Day 35. (Scale bars: 500 μm) (D) Gene expression of airway epithelial cell markers determined by qRT-PCR normalized to that of human adult lung. (E) Immunofluorescent imaging on Day 35. Red, p63; Gray, EpCAM; Blue, nuclei (Hoechst). (Scale bars: 50 μm) (F) Gene expression of AT2 cell markers by qRT-PCR normalized to that of human adult lung.

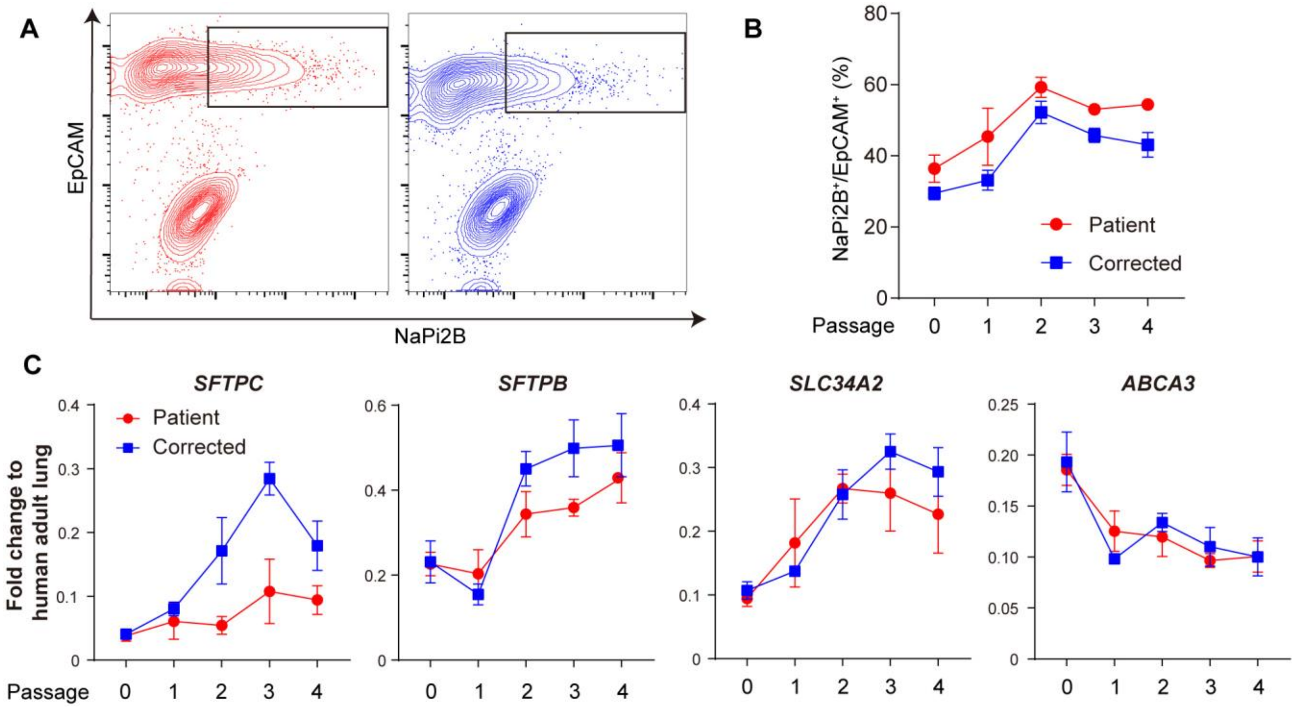

Fig. S5 Passage of alveolar organoids (AOs) using NaPi2B as a surface antigen, related to Fig. 3

(A) Gating on flow cytometry for passaging AOs. Representative flow cytometry plots at passage 0 of HPS1 iPSC-derived AOs and their gene-corrected counterparts are shown. (B) Ratio of NaPi2B$^+$ cells in EpCAM$^+$ cells during the periodic passaging of AOs. Data are presented as mean ± SEM (n = 3 from 3 independent experiments). (C) AT2 marker gene expression during the periodic passaging of AOs determined by qRT-PCR, normalized to that of human adult lung. Data are presented as mean ± SEM (n = 3 from 3 independent experiments).

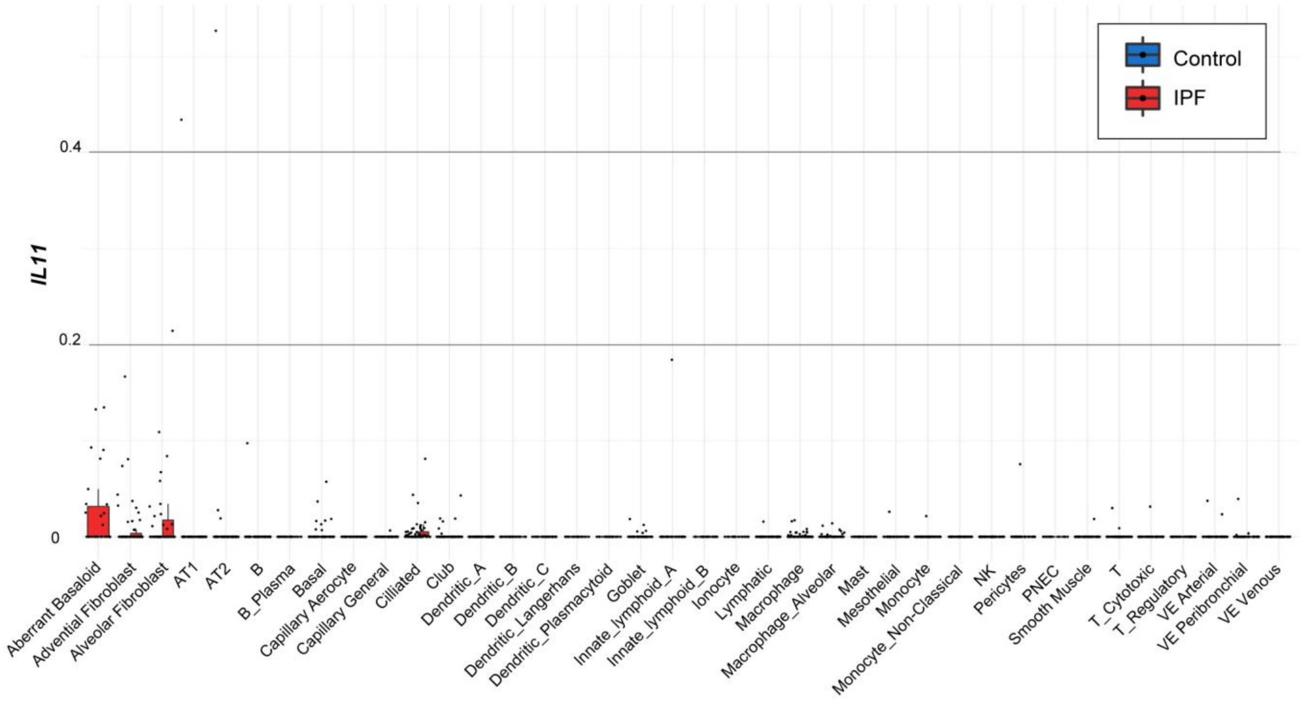

Fig. S6 Analysis of *IL11*-expressing cells using the IPF cell atlas, related to Fig. 4

We used Kaminski/Rosas data set (www.ipfcellatlas.com).

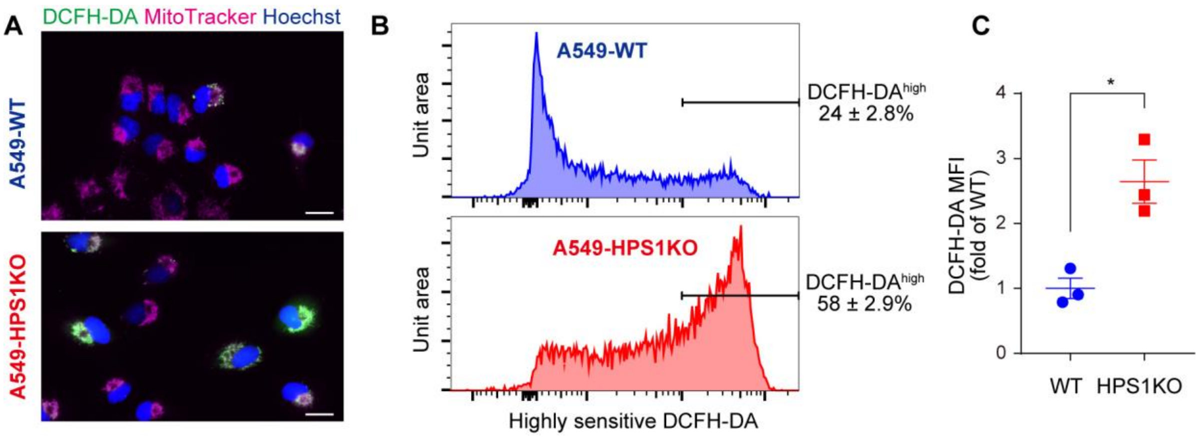

Fig. S7 Validation of phenotypes observed in patient-specific AOs using HPS1 KO A549 cells, related to Fig. 5

(A) Live cell imaging of intracellular reactive oxygen species (ROS) of HPS1 KO A549 cells using DCFH-DA dye and MitoTracker Deep Red FM. (Scale bars: 20 μm) (B and C) Quantification of DCFH-DA

staining intensity in HPS1 KO A549 cells by flow cytometry. Data are presented as mean ± SEM (n = 3 from 3 independent experiments). Unpaired two-tailed Student's t test: **P* < 0.05. MFI: Mean Fluorescence Intensity.

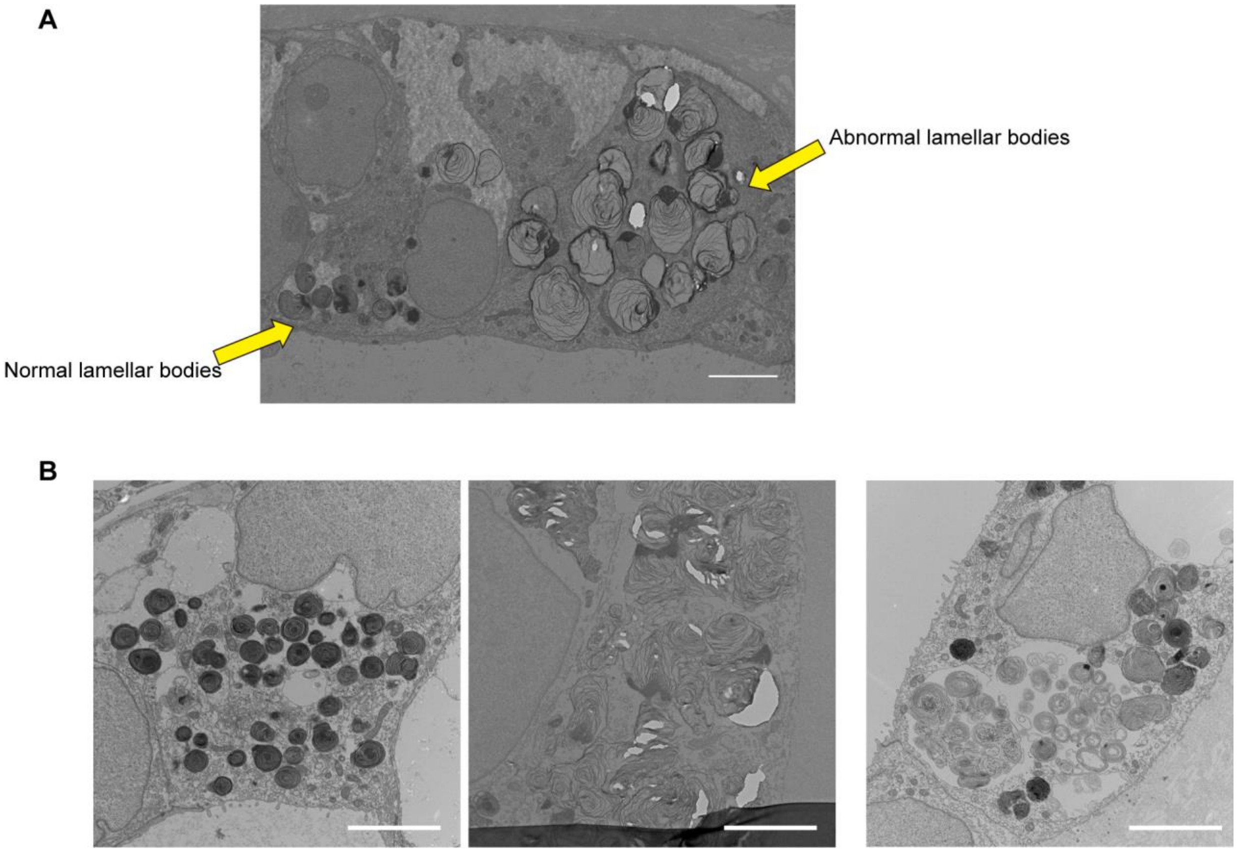

Fig. S8 Heterogeneous lamellar bodies of HPS1 patient-specific alveolar organoids (AOs), related to Fig. 5

Transmission electron microscope images of lamellar bodies in patient-specific AOs. (Scale bars: 5 μm) (A) Both abnormal and normal-shaped lamellar bodies were present in HPS1 patient-specific alveolar epithelial cells in organoids. (B) Miscellaneous lamellar body structures in HPS1 patient-specific AOs: left, normal; center, sparsely expanded lamellar structure but not spherical; right, excessively fused lamellar bodies.

SUPPLEMENTAL TABLES

Table S1 Primary antibodies used in the present study

**Host**	**Epitope**	**Supplier**	**Product**	**Permeabilization**	**Dilution**	**Dilution buffer**
Immunofluorescence of undifferentiated iPSCs in 2D culture						
Mouse	NANOG	Santa Cruz Biotechnology	sc-21704	0.2% Triton/ PBS	1:100	1%BSA/5% donkey serum/PBS
Rat	SOX2	Thermo Fisher Scientific	14-9811-80		1:500	
Mouse	OCT3/4	Santa Cruz Biotechnology	sc-5279		1:100	
Mouse	SSEA4	Santa Cruz Biotechnology	sc-293121		1:100	
Live cell flow cytometry						
Mouse	IgM isotype control	Sigma-Aldrich	M5909		1:100	1%BSA/10 μM Y-27632/PBS
Mouse	TRA1-60	Santa Cruz Biotechnology	sc-21705		1:100	
Mouse	THY1-FITC	Thermo Fisher Scientific	11-0909-41		1:50	
Mouse	CPM	Fujifilm Wako	014-27501		1:100	
Mouse	EpCAM-APC	Miltenyi Biotec	130-113-260		1:100 - 1:200	
Goat	EpCAM	Bio-Techne	AF960		1:100	
Mouse	NaPi2B	kindy provided by Dr. Gerd Ritter (MX35)			1:100	
Intracellular flow cytometry						

Mouse	CPM	Fujifilm Wako	014-27501	Methanol	1:100	1%BSA/10 µM Y-27632/PBS
Goat	EpCAM	Bio-Techne	AF960		1:100	
Rabbit	NKX2-1	Abcam	ab76013		1:100	
Magnetic-activated cell sorting						
Mouse	CPM	Fujifilm Wako	014-27501		1:100	1%BSA/10 µM Y-27632/PBS
Mouse	EpCAM	Santa Cruz Biotechnology	sc-66020		1:100	
Immunofluorescence of C-LBOs						
Rabbit	NKX2-1	Abcam	Ab76013	0.2% Triton/ PBS	1:100	1%BSA/5% donkey serum/PBS
Rat	SOX2	Thermo Fisher Scientific	14-9811-80		1:500	
Rabbit	SOX9	Abcam	ab185230		1:500	
Goat	EpCAM	Bio-Techne	AF960		1:100	
Rabbit	p63	Cell Signaling Technology	13109		1:500	
Immunofluorescence of AOs						
Mouse	NaPi2B	kindly provided by Dr. Gerd Ritter (MX35)		Methanol	1:100	Can Get Sigmal immunostain Solution B (Toyobo)
Rabbit	SPC	Seven Hills Bioreagents	WRAB-9337		1:100	
Mouse	ABCA3	Seven Hills Bioreagents	WMAB-ABCA3-17		1:100	
Goat	EpCAM	Bio-Techne	AF960		1:500	
Immunoblotting						
Mouse	β-Actin	Sigma-Aldrich	A2228		1: 5000	3%BSA/TBS-T
Rabbit	HPS1	Proteintech	15077-1-AP		1:400	

Rabbit	HPS4	Proteintech	14627-1-AP		1:400	Can Get Sigmal Solution 1 (Toyobo)

Table S2 Secondary antibodies used in the present study

**Host**	**Epitope**	**Conjugation**	**Supplier**	**Product**	**Dilution**
Donkey	Mouse IgG	Alexa Fluor 488	Thermo Fisher Scientific	A-21202	1:500
Donkey	Mouse IgG	Alexa Fluor 546	Thermo Fisher Scientific	A-10036	1:500
Donkey	Mouse IgG	Alexa Fluor 647	Thermo Fisher Scientific	A-31571	1:100 - 1:500
Donkey	Rabbit IgG	Alexa Fluor 546	Thermo Fisher Scientific	A-10040	1:500
Donkey	Rabbit IgG	Alexa Fluor 647	Thermo Fisher Scientific	A-31573	1:500
Donkey	Goat IgG	Alexa Fluor 488	Thermo Fisher Scientific	A-11055	1:500
Donkey	Goat IgG	Alexa Fluor 647	Thermo Fisher Scientific	A-21447	1:500
Donkey	Rat IgG	DyLight 550	Thermo Fisher Scientific	SA5-10027	1:100
Rat	Mouse IgM	APC	BioLegend	406509	1:200
Goat	Mouse IgG	MicroBeads	Miltenyi Biotec	130-048-401	1:5
Sheep	Mouse IgG	HRP	Cytiva	NA931V	1:5000
Donkey	Rabbit IgG	HRP	Cytiva	NA934V	1:5000

Table S3. Primers for TaqMan qPT-PCR

**Gene name**	**Taqman ID**
18S rRNA	Hs99999901_s1
EPCAM	Hs00901885_m1
TGFB1	Hs99999918_m1
SNAI2	Hs00950344_m1

**Gene name**	**Taqman ID**
HPS1	Hs00945778_m1
CDH1	Hs01023895_m1
ACTA2	Hs00426835_g1

Table S4. Primers for SYBR green qRT-PCR

Gene name	Forward primer sequence	Reverse primer sequence
18S rRNA	TTGACGGAAGGGCACCACCAG	GCACCACCACCCACGGAATCG
NKX2-1	AGCACACGACTCCGTTCTC	GCCCACTTTCTTGTAGCTTTCC
VIM	CGGGAGAAATTGCAGGAGGA	AAGGTCAAGACGTGCCAGAG
SOX2	GCACATGAAGGAGCACCCGGATTA	CGGGCAGCGTGTACTTATCCTTCTT
SOX9	GAGGAAGTCGGTGAAGAACG	ATCGAAGGTCTCGATGTTGG
IL11	GGACCACAACCTGGATTCCCTG	AGTAGGTCCGCTCGCAGCCTT
SNAI1	AATCCAGAGTTTACCTTCCAGCA	TCCCAGATGAGCATTGGCAG
PDGFRB	CGTCAAGATGCTTAAATCCACAGC	TGATGATATAGATGGGTCCTCCTTTG
THY1	ATCTCCTCCCAGAACGTC	ATCTCTGCACTGGAACTTG
COL1A1	CCCCGAGGCTCTGAAGGTC	GGAGCACCATTGGCACCTTT
FN1	AAGCAAGCCCGGTTGTTATG	AAACCAACGCATTGCCTAGG
TP63	ACTGCCAAATTGCAAAGACA	TGACTAGGAGGGGCAATCTG
KRT5	GAGCTGAGAAACATGCAGGA	TCTCAGCAGTGGTACGCTTG
SPDEF	AAGTGCTCAAGGACATCGAGA	AGGAGCCACTTCTGCACATT
MUC5AC	CATCTGCCAGCTGATTCTGA	AAGACGCAGCCCTCATAGAA
SCGB1A1	CACCATGAAACTCGCTGTCAC	AGTTCCATGGCAGCCTCATAAC
SCGB3A2	CAAGTGGAACCACTGGCTTG	CCAGAGGTAAAGGTGCCAAC
FOXJ1	CCTGTCGGCCATCTACAAGT	AGACAGGTTGTGGCGGATT
SNTN	GCTGCAAACCCAATTTAGGA	TGCTCATCAAGTTCAGAAAGGA
SFTPB	GAGCCGATGACCTATGCCAAG	AGCAGCTTCAAGGGGAGGA
SFTPC	GCAAAGAGGTCCTGATGGAG	TGTTTCTGGCTCATGTGGAG
ABCA3	TCTCCTTCAGCTTCATGGTCAG	TGGCTCAGAGTCATCCAGTTG
SLC34A2	TCGCCACTGTCATCAAGAAG	CTCTGTACGATGAAGGTCATGC

Table S5 (separate file). RNA-seq processed data of NaPi2B^{high} cells isolated from alveolar organoids.

Table S6 (separate file). RNA-seq processed data of EpCAM^{+} cells isolated from lung bud organoids, reanalysis of the publicly available data (GSE121999).

Table S7 (separate file). RNA-seq processed data of each model on lung epithelial cell marker gene expression.

Table S8 (separate file). Proteomic data of EpCAM^{+} cells isolated from alveolar organoids.

Table S9. Summary of the organoid model.

	LBOs	C-LBOs	AOs
**Authors**	Chen et al., *Nat Cell Biol*. 2017; Strikoudis et al., *Cell Rep*. 2019	This paper	Yamamoto et al., *Nat Methods*. 2017; Korogi et al., *Stem Cell Reports*. 2019
**Enrichment of NKX2-1^{+} lung progenitor**	No	Yes<br>By surface expression of CPM	Yes<br>By surface expression of CPM
**Organoid formation methods**	Spontaneous folding on flat-bottomed low-adherent plate	Forced aggregation on U-bottomed low-adherent plate	Co-culture with primary lung fibroblasts
**Medium component in Matrigel phase**	CHIR99021;<br>BMP4;<br>KGF;<br>FGF10;<br>ATRA	CHIR99021;<br>BMP4;<br>KGF;<br>FGF10;<br>ATRA	Dexamethasone;<br>Cyclic AMP;<br>IBMX;<br>KGF
**Organoid morphology**	Branching structure	Branching structure	Spheroidal structure
**Transcriptome of epithelial cells**	Maintenance of NKX2-1;<br>Transcriptome of LBOs are matched to that of lungs in the second trimester of human gestation	Loss of NKX2-1;<br>Not specified	Maintenance of NKX2-1;<br>SPC^{+} cells in AOs are similar to Sftpc^{+} cells from E18.5 mouse
**AT2 cell marker expression**	Low	No	High
**Mesenchymal cell present**	Yes; minimal	No	Yes; co culture with primary fibroblasts
**Phenotypes of HPS1-deficiency**	Abnormal morphology;<br>High *IL11* expression;<br>Enhanced proliferation of mesenchymal cells	Abnormal morphology;<br>High *IL11* expression;<br>Partial EMT;<br>Upregulation of *TGFB1*	Giant lamellar body;<br>Mitochondrial dysfunction
